# Ultrafast Interfacial Self‐Assembly toward Supramolecular Metal‐Organic Films for Water Desalination

**DOI:** 10.1002/advs.202201624

**Published:** 2022-07-03

**Authors:** Zhao Zhang, Chang Liu, Huilin Zhang, Zhi‐Kang Xu, Feng Ju, Chengbing Yu, Yuxi Xu

**Affiliations:** ^1^ School of Engineering Westlake University, Westlake Institute for Advanced Study 18 Shilongshan Road Hangzhou Zhejiang Province 310024 China; ^2^ MOE Key Laboratory of Macromolecular Synthesis and Functionalization Key Laboratory of Adsorption and Separation Materials & Technologies of Zhejiang Province Department of Polymer Science and Engineering Zhejiang University Hangzhou 310027 China; ^3^ Key Laboratory of Coastal Environment and Resources of Zhejiang Province, School of Engineering Westlake University 18 Shilongshan Road Hangzhou Zhejiang Province 310024 China; ^4^ School of Materials Science and Engineering Shanghai University Shanghai 201800 China

**Keywords:** 1,3,5‐Triformylphloroglucinol, coordination interaction, interfacial assembly, supramolecular metal‐organic film, water desalination

## Abstract

Supramolecular metal‐organic materials are considered as the ideal candidates for membrane fabrication due to their excellent film forming characteristics, diverse metal centers and ligand sources, and designable structure and function. However, it remains challenging to rapidly construct highly permeable supramolecular metal‐organic membranes with high salt rejection. Herein, a novel ultrafast interfacial self‐assembly strategy to prepare supramolecular metal‐organic films through the strong coordination interaction between highly active 1,3,5‐triformylphloroglucinol (TFP) ligands and Fe^3+^, Sc^3+^, or Cu^2+^ at the organic–aqueous interface is reported. Benefiting from the self‐completing and self‐limiting characteristics of this interfacial self‐assembly, the new kind of supramolecular membrane with optimized composition can be assembled within 3.5 min and exhibits ultrathin, dense, defect‐free features, and thus shows an excellent water permeance (21.5 L m^–2^ h^–1^ bar^–1^) with a high Na_2_SO_4_ rejection above 95%, which outperforms almost all of the non‐polyamide membranes and commercially available nanofiltration membranes. This strong‐coordination interfacial self‐assembly method will open up a new way for the development of functional metal‐organic supramolecular films for high‐performance membrane separation and beyond.

## Graphical abstract

A new ultrafast coordination‐driven interfacial assembly strategy is reported to construct ultrathin dense supramolecular metal‐organic nanofilms via strong coordination interaction between metal ions (Fe^3+^, Sc^3+^, Cu^2+^) and a highly active polyphenol (1,3,5‐triformylphloroglucinol) at the water/oil interface, which shows excellent separation performance for water desalination.

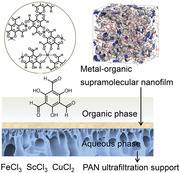

## 1 Introduction

Membrane‐based water purification technologies have attracted widespread attention due to their low energy consumption, continuous operation, and environmental friendliness.^[^ ^1^, ^2^, ^3^, ^4^ ^]^ In order to achieve high‐efficient separation, highly permeable and selective membranes are extremely desired. Taking advantage of excellent film forming characteristics, diverse metal centers and ligand sources, and tunable pore structures and functions,^[^ ^5^, ^6^ ^]^ supramolecular metal‐organic materials hold promising candidates for constructing high‐performance membranes. So far, crystalline and amorphous metal‐coordinated materials represent two kinds of most studied supramolecular metal‐organic materials. Over the past decade, crystalline metal organic framework (MOF) membranes that exhibit inherently uniform and ordered sub‐nanometer pores, which just located between the diameters of water molecules (0.28 nm) and common hydrated ions (≥0.66 nm), have been widely applied in water–salt separation.^[^ ^7^, ^8^ ^]^ Up to now, a series of continuous MOF membranes (like UiO‐66,^[^ ^9^ ^]^ MOF‐303,^[^ ^10^ ^]^ ZIF‐8,^[^ ^11^ ^]^ etc.) have been fabricated through solvothermal synthesis or layer‐by‐layer self‐assembly and showed high rejections toward multivalent ion (≥90% for Na_2_SO_4_). However, to avoid the formation of grain boundary defects and intercrystalline cracks, the synthesized MOF membranes were relatively thick and thus resulted in a low permeance (0.15–5.0 L m^–2^ h^–1^ bar^–1^), which were inferior than commercial polyamide membranes. In addition, the long film‐formation time and the difficulty in the production of large‐area (>1 m^2^) and defect‐free films also greatly limit the development and application of MOF membranes.^[^ ^12^, ^13^ ^]^

Compared with crystalline MOF membranes, amorphous metal‐organic materials are supposed to form the ultrathin nanofilms without defects. Since the discovery of one‐step self‐assembly between tannic acid (TA) and Fe^III^ ion in 2013,^[^ ^14^ ^]^ the amorphous metal‐phenolic supramolecular networks have become a hotspot of research in membrane preparation because of their excellent film‐forming feasibility, stable cross‐linked structures and tunable pore size.^[^ ^15^, ^16^ ^]^ In the past years, such amorphous metal‐coordinated membranes have been demonstrated to exhibit ultrafast water transport and high dye rejection.^[^ ^17^, ^18^, ^19^, ^20^ ^]^ Unfortunately, due to the water‐soluble characteristics of organic ligands (such as tannic acid, phytic acid, etc.), most of the amorphous supramolecular membranes are fabricated through time‐consuming aqueous self‐assembly with relatively loose cross‐linked structures and large pore sizes, making it difficult to achieve high‐efficient water–salt separation. Therefore, it is highly desired to develop novel strategy for the rapid construction of ultrathin and dense supramolecular metal‐organic membrane with both ultrahigh water permeance and water–salt selectivity.

Herein, we report a simple, ultrafast coordination‐driven interfacial self‐assembly strategy to prepare supramolecular metal‐organic film at water/o‐xylene interface. 1,3,5‐triformylphloroglucinol (TFP) was chosen as the organic ligand because its strong chelating ability of O‐based hexadentate ligand could enable the ultrafast formation of highly cross‐linked network structure with metal ions. Moreover, the oil‐soluble and hydrophobic characteristics of TFP was beneficial to construct strictly organic–aqueous interfacial self‐assembly system with a variety of metal ions‐containing (including Fe^3+^, Sc^3+^, Cu^2+^) aqueous phase, allowing the formation of supramolecular film at water/o‐xylene interface. It was believed that the self‐completing and self‐limiting characteristics of this interfacial assembly could endow the metal‐organic film with continuous, ultrathin and defect‐free characteristics, which are considered as promising candidates for membrane separation. Thus, we constructed supramolecular separation membranes on polyacrylonitrile substrate through in situ self‐assembly and the obtained composite membrane exhibited a high permeance with excellent salt rejection. In addition, it was noting that such in situ ultrafast interfacial assembly strategy showed excellent scale‐up capability through the integration within a typical roll‐to‐roll processing system, which was also applied in the preparation of traditional commercial polyamide composite membrane.^[^ ^21^ ^]^

## 2 Results and Discussion

**Figure 1:**
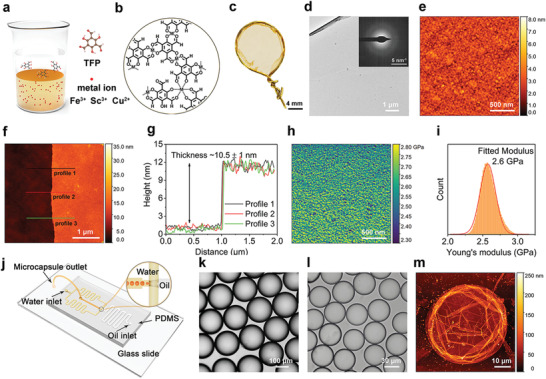
Description of the supramolecular interfacial self‐assembly process and the resulting nanofilms and microcapsules. a) Schematic representation of the interfacial self‐assembly process used to synthesize the metal‐TFP nanofilms, the bottom yellow layer is the metal ions‐containing aqueous solution, and the top colorless layer corresponds to TFP in o‐xylene. b) The possible cross‐linked network structure of the resultant metal‐TFP nanofilm. c) Photograph of the ultrathin Fe^3+^‐TFP nanofilm transferred to a wire lasso. d) TEM image of the Fe^3+^‐TFP nanofilm. Inset, the corresponding SAED pattern). e) AFM topography image, f) AFM height image, and g) corresponding height profile of Fe^3+^‐TFP nanofilms transferred onto silicon wafers. h) Young's modulus mapping and i) the corresponding statistical histogram of the Fe^3+^‐TFP nanofilm transferred onto the silicon wafer. j) Schematic representation of the microcapsules generation process using a microfluidic device, consisting of a TFP‐containing o‐xylene (oil) phase perpendicular to a continuous aqueous solution containing metal ions. Optical micrographs of the highly monodisperse and sized‐controlled Fe^3+^‐TFP microcapsules formed with different flow rates: k) 1.5 µL min^–1^ for oil phase and 3 µL min^–1^ for water phase, and l) 10 µL min^–1^ for oil phase and 20 µL min^–1^ for water phase. m) The corresponding AFM image of the dried microcapsule from (l).

**Figure** **1**a illustrates the interfacial self‐assembly for the formation of metal‐TFP nanofilms with highly cross‐linked network structure (Figure 1b). Typically, metal salt was dissolved in water to form aqueous solution and placed at the bottom of a beaker, and then, TFP‐containing o‐xylene solution was carefully poured onto the water phase. Once the o‐xylene phase came into contact with water phase, there would be a continuous and ultrathin nanofilm formed immediately at the water/o‐xylene interface. The resulting metal‐TFP nanofilms could be readily transferred onto any substrates. Figure 1c exhibits a smooth Fe^3+^‐TFP nanofilm transferred to a wire lasso; although the nanofilm was ultrathin, it formed an integral, defect‐free surface across the lasso with a diameter of 1.3 cm, indicating the good robustness of the nanofilm. Under optical microscope (Figure S1, Supporting Information), the Fe^3+^‐TFP nanofilm exhibited homogeneous bright blue contrast on SiO_2_/Si substrate (light purple area). Further transmission electron microscopy (TEM) and selected area electron diffraction (SAED) analysis (Figure 1d, Figure S2, Supporting Information) demonstrated the amorphous nature of Fe^3+^‐TFP nanofilm. For morphological and mechanical characterization, the freestanding nanofilms coordinated with different metal ions were carefully transferred to silicon wafer and atomic force microscope (AFM) analysis was performed. It can be seen that all the nanofilm surfaces coordinated with different metal ions (Fe^3+^, Sc^3+^, and Cu^2+^) were composed of many small globular‐like nodular structures, and the nanofilm thickness was ≈10 nm (Figure 1e–g, Figures S4a–c and S5a–c, Supporting Information). Interestingly, such 10 nm‐thick metal‐TFP nanofilms possessed Young's moduli in the range of 2.1 to 3.0 GPa (Figure 1h,i, Figures S4d,e and S5d,e) depending on the different metal ions, showing surprising mechanical strengths. Considering the versatility of interfacial self‐assembly, we also tried the microcapsule fabrication in one step by using a microfluidic platform. As shown in Figure 1j, the TFP‐containing o‐xylene (oil) phase intersected with the perpendicularly flowing continuous aqueous phase composed of FeCl_3_ and poly(vinyl alcohol), leading to droplets of o‐xylene periodically being sheared at the microfluidic flow‐focusing junction. With a flow ratio of 2:1 of the water phase to the o‐xylene phase, stable and uniform microcapsules were continuously generated. Moreover, the size of microcapsules could be tuned from 205 to 42 µm by adjusting the water/o‐xylene flow rate (Figure 1k,l). AFM analysis (Figure 1m, Figure S7, Supporting Information) showed that the single‐wall thickness of the capsule was supposed to be half of the minimum thickness of the dried microcapsule (≈12.5 nm), which was close to the thickness of freestanding metal‐TFP nanofilms. These template‐free, size‐controlled supramolecular microcapsules could effectively entrap guest species, and, in the future, could be applied in the field of drug delivery.

**Figure 2:**
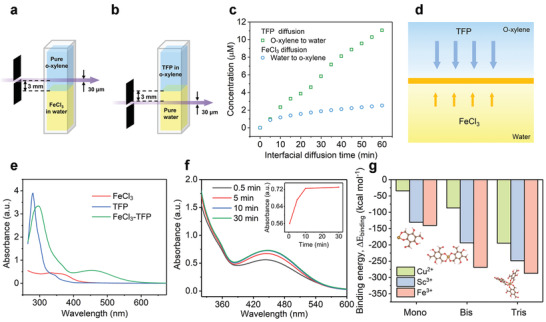
Interfacial diffusion kinetics of the monomers and coordination interaction between TFP and metal ions. Schematic presentation of in situ monitoring of interfacial diffusion by UV–vis spectroscopy. a) Monitoring FeCl_3_ (5 mm) diffusion from water phase to pure o‐xylene phase. b) Monitoring TFP (5 mm) diffusion from o‐xylene phase to pure water phase. c) Monomer concentration detected at a position 3 mm away from the water/o‐xylene interface versus interfacial diffusion time via UV–vis spectroscopy‐based in situ monitoring as shown in (a) and (b). d) Schematic diagram of the monomer diffusion direction at water/o‐xylene interface. e) UV–vis spectra of TFP, FeCl_3_, and their complex. f) Time‐dependent UV–vis spectra changes of the assembled Fe^3+^‐TFP complex, inset, time‐dependent absorbance changes at 445 nm (LMCT band). g) Calculated binding energy (Δ*E*_binding_, kJ cal^−1^) between TFP and different metal ions.

It is well known that interfacial polymerization is a complex reaction–diffusion process far from thermodynamic equilibrium, where the monomer diffusion kinetics could have important effects on the reaction progress and film formation at the water/oil interface.^[^ ^22^, ^23^ ^]^ To explore the FeCl_3_ and TFP diffusion mechanism across the water/o‐xylene interface, we carried out the diffusion kinetics tests of FeCl_3_ and TFP across the water/o‐xylene interfaces in real‐time with UV–vis spectroscopy (**Figure** **2**a,b). It was clear from Figure 2c that the diffusion rate of TFP from o‐xylene into water was similar to that of FeCl_3_ from water into o‐xylene at initial 5 min, and then TFP diffusion still quickly proceeded while FeCl_3_ diffusion gradually achieved maximum. To explain such different diffusion behavior, the partition coefficients (log*P*_A/B_) of FeCl_3_ and TFP between water and o‐xylene were obtained through the density functional theory calculations.^[^ ^24^ ^]^ The results showed that the log*P* value of FeCl_3_ in the cases of water/o‐xylene is 8.35, while the log*P*_o‐xylene/water_ value of TFP was 3.14 (Table S1, Supporting Information). The almost twofold relation implied that FeCl_3_ was far more difficult to diffuse into the o‐xylene phase than TFP into water phase (Figure 2d), leading to a continuously increasing TFP concentration in water phase and a slow increase of FeCl_3_ concentration in o‐xylene phase. Based on these results, we proposed a diffusion–reaction model describing the coordination‐driven interfacial assembly process. That was, TFP was more willing to diffuse from the o‐xylene phase into the water phase and coordinated with Fe^3+^ to form a dense supramolecular film and the Fe^3+^‐TFP nanofilm mainly grew along the same direction of TFP diffusion into the water phase rather than into the o‐xylene phase.

In order to study the coordination reaction mechanism and the inherent metal–ligand interaction, the complexation behavior between TFP and different metal ions in ethanol was monitored by UV–vis absorption spectroscopy. As shown in Figure 2e,f, the characteristic ligand‐to‐metal charge transfer (LMCT) band of Fe^3+^‐TFP complexes appeared at ≈445 nm, the intensity of which was found to increase as a function of time and achieved saturation after 10 min, implying complete complexation of Fe^3+^ with TFP. This short saturation time demonstrated the strong chelating ability of TFP with metal ions, which enabled the formation of supramolecular metal‐organic nanofilms in a few seconds. Compared with Fe^3+^‐TFP complexes, the LMCT band of Sc^3+^‐TFP and Cu^2+^‐TFP was not very apparent and appeared at ≈375 nm (Figure S8, Supporting Information). These differences in peak position and intensity of LMCT bands reflected the binding strength of TFP with different metal ions. For better understanding the coordination intensity of different metal ions, density functional theory (DFT) calculation was conducted to obtain the binding energy of metal‐TFP complexes. As shown in Figure 2g, the binding energy of mono‐complexes varied from −34.3 kcal mol^−1^ for Cu^2+^, to larger negative values of –130.7 kcal mol^−1^ for Sc^3+^ and −140.5 kcal mol^−1^ for Fe^3+^, indicating the increasing coordination interaction. Moreover, the binding energy of bis‐ and tris‐complexes also followed sequence Cu^2+^ < Sc^3+^ < Fe^3+^, which was consistent with UV–vis results. In addition, the metal–ligand interactions in metal‐TFP nanofilms were further probed by attenuated total reflectance infrared spectroscopy (ATR‐IR) spectroscopy and Raman spectroscopy. ATR‐IR spectra (Figure S10, Supporting Information) of the metal‐TFP nanofilms indicated that TFP was strongly coordinated with metal ions, as evidenced by the new peak of metal–oxo stretching mode^[^ ^25^ ^]^ appeared at 523 cm^–1^, when compared with non‐coordinated TFP. The Raman spectrum (Figure S11, Supporting Information) also revealed the Fe–O vibration in the low‐frequency region of 600–400 cm^−1^. The elemental distribution was investigated by high‐angle annular dark‐field scanning transmission electron microscope (HAADF‐STEM) and energy dispersive X‐ray spectroscopy (EDX) elemental maps (Figure S12, Supporting Information), which showed that C, O and Fe were uniformly distributed in the resultant Fe^3+^‐TFP nanofilms. Furthermore, the X‐ray photoelectron spectroscopy (XPS) spectra (Figure S13, Supporting Information) also confirmed the presence of metal ions in the nanofilms.

**Figure 3:**
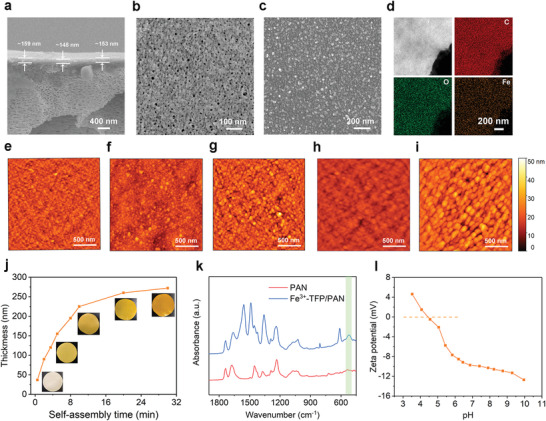
Metal‐TFP composite membrane characterizations. a) Cross‐sectional SEM image of Fe^3+^‐TFP/PAN composite membrane with a self‐assembled time of 5 min. Inset, the high magnification image. b) Surface SEM images of the PAN substrate and c) Fe^3+^‐TFP membrane formed on the PAN substrate with a self‐assembled time of 5 min. d) HAADF‐STEM image and elemental maps of the isolated Fe^3+^‐TFP active layer with a self‐assembled time of 5 min. AFM images of isolated Fe^3+^‐TFP membranes with different self‐assembly time: e) 0.5 min, f) 3.5 min, g) 5 min, h) 10 min, i) 30 min. j) Fe^3+^‐TFP layer thickness as a function of self‐assembly time, which were obtained from AFM height profiles. k) ATR‐IR spectra of Fe^3+^‐TFP/PAN composite membrane and PAN substrate. l) Zeta potential of Fe^3+^‐TFP/PAN composite membrane.

Given that the interfacial self‐assembled metal‐TFP nanofilm exhibited dense structures with ultrathin and defect‐free features, they were considered as attractive candidates for preparing ultrafast and highly selective membranes for separation applications. Thus, in the following experiment, we constructed metal‐TFP composite membrane through in situ interfacial self‐assembly strategy on polyacrylonitrile (PAN) substrate. Such in situ assembly strategy was believed to effectively improve the adhesive strength the between metal‐TFP nanofilms and PAN substrate, ensuring enough robustness under practical cross‐flow operation conditions. It should be noted that PAN ultrathin membrane was select as the substrate due to its excellent tolerance to o‐xylene, while other polymeric membrane such as polysulfone and polyethersulfone would be partly dissolved in o‐xylene. As exhibited in **Figure** **3**a–c, it was obvious that the self‐assembled membrane was constituted by a relatively dense thin film (≈155 nm) on the top of a porous support membrane, implying the successful formation of the Fe^3+^‐TFP active layer on PAN substrate. ATR‐IR spectra (Figure 3k) also demonstrated the generation of Fe^3+^‐TFP layer, as evidenced by the appearance of new Fe‐O stretching peak at 523 cm^–1^. EDX mapping analysis of the isolated Fe^3+^‐TFP layer indicated that Fe distribution pattern fairly matched with the HAADF image and the distribution patterns of C and O maps (Figure 3d). This result demonstrated that Fe^3+^ was coordinated with TFP and uniformly distributed in the Fe^3+^‐TFP layer, and the participation of PAN substrate did not change the compositional homogeneity of Fe^3+^‐TFP layers. More interestingly, it was found from surface AFM images (Figure 3e–i) that the size of the surface globular‐like structure of Fe^3+^‐TFP layers gradually increased as the self‐assembled time prolonged from 0.5 to 10 min, implying the more cross‐linked and denser structure of Fe^3+^‐TFP layer. Meanwhile, the thickness of Fe^3+^‐TFP layer also increased from ≈37 to ≈220 nm (Figure 3j, Figure S14, Supporting Information), together with an improved Young's modulus from 3.3 to 6.6 GPa (Figure S15, Supporting Information). Further prolongation in assembly time was found to result in a slow increase of membrane thickness, demonstrating the self‐completing and self‐limiting characteristics of the metal‐coordinated interfacial assembly. Surface charge characteristics of the Fe^3+^‐TFP/PAN composite membrane was further determined by zeta potential measurement. As shown in Figure 3l, the composite membrane was negatively charged at pH of 7.0 and its isoelectric point was 4.5. This may be resulted from the dissociation of uncoordinated phenolic hydroxyl groups of TFP on the Fe^3+^‐TFP layer surface. It was believed that such negatively charged feature was conducive to improving the anions rejection through Donnan effect.^[^ ^26^ ^]^

**Figure 4:**
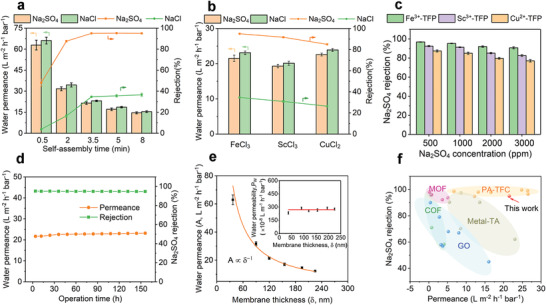
Separation performance of metal‐TFP composite membranes. a) Water permeance and salt rejection of Fe^3+^‐TFP/PAN composite membranes with varied self‐assembly time, FeCl_3_, and TFP concentration were fixed at 5 and 6 mm, respectively. b) Water permeance and salt rejection of metal‐TFP/PAN composite membranes with different metal ions. c) Na_2_SO_4_ rejection of metal‐TFP/PAN composite membrane as a function of N_2_SO_4_ concentration. d) Long‐term stability of Fe^3+^‐TFP/PAN composite membrane. e) Water permeance, *A*, as a function of Fe^3+^‐TFP active layer thickness, *δ*. Inset, thickness‐normalized water permeability, *P*_w_. f) Filtration performance comparison of optimal Fe^3+^‐TFP/PAN composite membrane with the state‐of‐the‐art membranes reported in previous literatures. Except for the special instruction in (c), other membranes were tested in a cross‐flow filtration system with 1000 ppm salt aqueous solution under 5.0 bar, 25 ± 2 °C and pH 7.5 ± 0.2.

**Figure 5:**
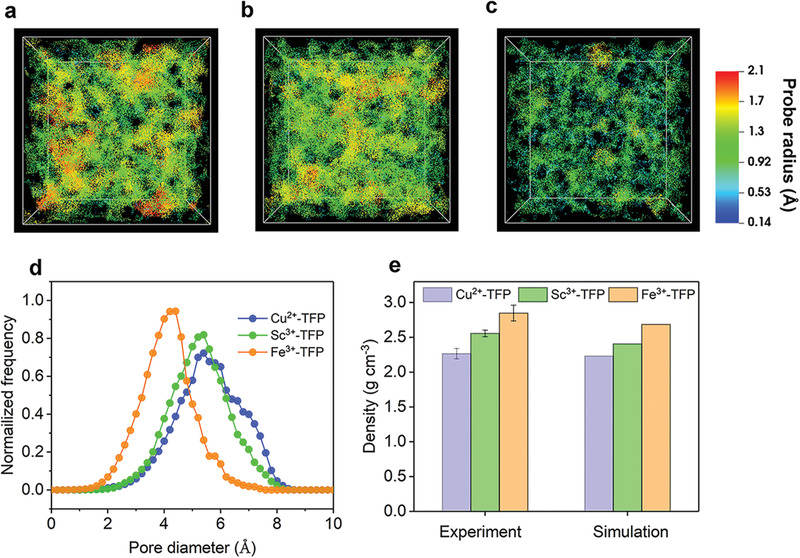
Structural analysis of amorphous polymer models. Coloring diagrams concerning the pore radius of a) Cu^2+^‐TFP membrane, b) Sc^3+^‐TFP membrane, and c) Fe^3+^‐TFP membrane. d) Simulated pore size distributions of metal‐TFP membranes. e) Experimentally measured density of metal‐TFP films versus simulated density values.

We then evaluated separation performance of supramolecular metal‐organic membranes through salt water filtration tests (**Figure** **4**, Figure S16, Supporting Information) and explored structure–property relations in these metal‐TFP/PAN membranes for water desalination. As shown in Figure 4a, when the self‐assembled time prolonged from 0.5 to 3.5 min, membrane salt rejection obviously increased from 48.1% to 95.0% for Na_2_SO_4_, and 3.9% to 34.7% for NaCl, combined with a sharp decrease in water permeance from 62.9 to 21.5 L m^–2^ h^–1^ bar^–1^. This reason may be that the cross‐linking degree between Fe^3+^ and TFP was improved with a longer reaction time and the assembled Fe^3+^‐TFP layer became denser, resulting in a higher salt rejection and lower water permeance. As the assembly time continued to increase from 3.5 to 10 min, the salt rejection kept nearly stable with a slight decrease in water permeance, implying the unchanged cross‐linking density but still an increase in Fe^3+^‐TFP film thickness. Note that the higher rejection of Na_2_SO_4_ than NaCl was a typical characteristic of negatively charged membrane, which was similar to the commercially piperazine‐based polyamide membrane and could be ascribed to the synergistic effect of size sieving and Donnan exclusion.^[^ ^27^, ^28^ ^]^ Interestingly, the supramolecular metal‐ TFP/PAN membranes utilizing Fe^3+^, Sc^3+^, and Cu^2+^ as coordinated metal ions showed different permselectivities (Figure 4b). The Fe^3+^ coordinated membrane exhibited the highest salt rejection, followed by Sc^3+^‐TFP membrane, and the salt rejection of Cu^2+^‐TFP membrane was the lowest. Meanwhile, the water permeances of three supramolecular membranes were relatively close and in the range of 19.3 to 22.6 L m^–2^ h^–1^ bar^–1^. To understand the performance difference of TFP coordinated membranes with different metal ions in‐depth, we performed molecular simulations to construct realistic structural models and analyze their porous structures. The amorphous polymer models were generated using Amorphous Cell module in Materials Studio packages. Details of the simulations were given in the supporting information. The voids colored with respect to the pore radius and the corresponding pore‐size distribution for each metal‐TFP model were shown in **Figure** **5**a–d. It could be seen that all the metal‐TFP membranes have pore sizes smaller than 8 Å and were very close to the hydrated diameter of salt ions (e.g., 7.6 Å for SO_4_^2–^, 7.0 Å for Na^+^ and 6.6 Å for Cl^–^),^[^ ^29^ ^]^ theoretically indicating the feasibility of desalination through these supramolecular metal‐organic membranes. Furthermore, the actual porous structure was demonstrated by X‐ray diffraction test (Figure S16, Supporting Information), the average chain d‐spacing (calculated by Bragg's law) indicated the sub‐nanoporous structural characteristics for Fe^3+^‐TFP (*d* = 5.72 Å), Sc^3+^‐TFP (*d* = 6.05 Å), and Cu^2+^‐TFP (6.16 Å), which was consistent with the simulated pore size sequence (Fe^3+^‐TFP < Sc^3+^‐TFP < Cu^2+^‐TFP) and the salt rejection behavior of Mn^+^‐TFP membranes. Importantly, it was found that the simulated density was very close to the experimental result (Figure 5e), and thus illustrating the reliability of our model. As shown Figure 4c, with increasing Na_2_SO_4_ concentration from 500 to 3000 ppm, the salt rejection maintained relatively stable, which meant that the metal‐TFP membranes were viable for concentrated salt solution.

Apart from water permeance and rejection, another key parameter to evaluate the membrane performance is the membrane operation stability. The long‐term filtration test of the Fe^3+^‐TFP/PAN membrane (Figure 4d) showed that excellent Na_2_SO_4_ rejection above 95% were realized even after 156 h with a slight increase in water permeance from 21.7 to 23.1 L m^–2^ h^–1^ bar^–1^. After filtration, the Fe^3+^‐TFP active layer surface remained the globular‐like structure, showing its good structural stability (Figure S19a, Supporting Information). On the other hand, the existence of Fe–O stretching peak at 523 cm^–1^ from ATR‐FTIR result also demonstrating its good coordination stability (Figure S19b, Supporting Information). Such excellent stability may be due to the strong coordination binding between Fe^3+^ and TFP as well as the high Young's modulus of Fe^3+^‐TFP active layer. Membrane thickness is usually related with the distance of molecule transport pathway through the membrane.^[^ ^17^ ^]^ To clarify the structure–performance relationship between membrane thickness and water transport capacity, water permeance with respect to Fe^3+^‐TFP layer thickness was presented in Figure 4e. We found that water permeance (*A*) was inversely proportional to the Fe^3+^‐TFP layer thickness (*δ*). After normalizing water permeance by their respective thicknesses, water permeability (*P*_w_ = A*δ) was determined to be 269.4 × 10^–8^ L m^–1^ h^–1^ bar^–1^ from the inset in Figure 4e. Compared with water permeance, water permeability as a thickness‐independent parameter was better to reflect the intrinsic water transport capability for a certain membrane material^[^ ^30^ ^]^ and the nearly constant water permeability suggested the homogeneous structure and composition of assembled Fe^3+^‐TFP layers. We further compared the water permeance and salt rejection of the Fe^3+^‐TFP membrane with the state‐of‐the‐art nanofiltration membranes reported in the literature (Figure 4f, Table S3, Supporting Information). It could be seen that the Fe^3+^‐TFP membrane exhibited an exceptional permeance and salt rejection, which outperformed most of the polyamide thin‐film‐composite (PA‐TFC) membranes and novel nanofiltration membranes prepared from metal‐organic framework (MOF), graphene oxide (GO), covalent organic framework (COF) and metal‐tannic acid (metal‐TA) based supramolecular materials reported in the previous literature. Therefore, the supramolecular Fe^3+^‐TFP membrane was expected to be an excellent candidate for water desalination.

## 3 Conculsion

In summary, we developed a new coordination driven interfacial self‐assembly strategy for the fabrication of supramolecular metal‐TFP films and microcapsules. The strong coordination interaction between metal ions and TFP enabled the fast formation of highly cross‐linked film structure at the water/o‐xylene interface. Benefiting from the ultrathin, robust and defect‐free characteristics, the supramolecular metal‐TFP films show their great potential in separation membrane construction for water purification. Therefore, composite membranes were successfully achieved by in situ interfacial self‐assembly on porous PAN support. The optimized Fe^3+^‐TFP/PAN membrane exhibited a high water permeance (21.5 L m^–2^ h^–1^ bar^–1^) with Na_2_SO_4_ rejection above 95%, which outperformed almost all of the state‐of‐the‐art membranes. This work will facilitate the development of new kinds of functional supramolecular metal‐organic films through rational molecular design for efficient membranes separation and beyond.

## Conflict of Interest

The authors declare no conflict of interest.

## Supporting information

Supporting Information

## Data Availability

The data that support the findings of this study are available from the corresponding author upon reasonable request.
