# Supplementary material for: Ultrafast Interfacial Self‐Assembly toward Supramolecular Metal‐Organic Films for Water Desalination
Source: Adv Sci (Weinh). 2022 Jul 3;9(24):2201624. doi: 10.1002/advs.202201624 (PMC9403643; doi:10.1002/advs.202201624)
Supplement: Supplementary file 1 — Supporting Information [file ADVS-9-2201624-s001.pdf]

ADVANCED SCIENCE
Open Access

Supporting Information

for *Adv. Sci.*, DOI 10.1002/advs.202201624

Ultrafast Interfacial Self-Assembly toward Supramolecular Metal-Organic Films for Water Desalination

Zhao Zhang, Chang Liu, Huilin Zhang, Zhi-Kang Xu, Feng Ju, Chengbing Yu and Yuxi Xu**

Supporting Information

Ultrafast interfacial self-assembly towards supramolecular metal-organic films for water desalination

Zhao Zhang, Huilin Zhang, Chang Liu, Feng Ju, Zhi-Kang Xu, Chengbing Yu, Yuxi Xu**

Materials

All reagents and solvents were commercially available and used as received. 1,3,5-triformylphloroglucinol (TFP) was purchased from Jilin Chinese Academy of Sciences-Yanshen Technology Co., Ltd. Iron chloride hexahydrate ($FeCl_3 \cdot 6H_2O$), scandium chloride hexahydrate ($ScCl_3 \cdot 6H_2O$), copper chloride dihydrate ($CuCl_2 \cdot 2H_2O$) and poly(vinyl alcohol) (87-90% hydrolyzed, average mol wt 30,000-70,000) were obtained from Sigma-Aldrich. O-xylene, dichloromethane, n-hexane, ethanol, N,N-dimethylformamide (DMF), propylene carbonate (PC), sodium chloride (NaCl), sodium sulfate (Na_2SO_4) were purchased from Sinopharm Chemical Reagent Co., Ltd. Polydimethysiloxane (PDMS, Sylgard 184) and its curing agent were obtained from Dow Coring (Midland, MI). Polyacrylonitrile (PAN) ultrafiltration membrane (MWCO = 50,000) was obtained by Beijing Separate Equipment Co., Ltd.

Methods

Fabrication of metal-TFP freestanding films

Metal-TFP freestanding films were synthesized in a glass beaker. First, 50 ml 5 mM metal salt ($FeCl_3$, $ScCl_3$, $CuCl_2$) aqueous solution was poured into the beaker, then 50 mL 5 mM 1,3,5-triformylphloroglucinol (TFP) of o-xylene was carefully added on top of the aqueous solution. Once contacted, there would be an ultrathin film formed immediately at the water/-o-xylene interface. After 20 s, the grown nanofilm was transferred onto a silicon wafer substrate or a copper grid by inserting substrates underneath of the interface in advance and

lifting the substrates up slowly. The resultant membrane was denoted as M^{n+}-TFP, where M^{n+} represented Fe^{3+}, Sc^{3+}, Cu^{2+}.

Fabrication of Microfluidic chips

The procedure of the master fabrication using digital light processing and microfluidic chip manufacture was demonstrated in detail in previous study. Briefly, the designed model was first printed by a commercial 3D printer (nanoArch® S140, Shenzhen, China). Then, the printed master was covered by polydimethysiloxane (PDMS) and its curing agent fully mixed at a ratio of 10:1 (w/w). The PDMS was cured at 80°C for 2 h and then peeled off and punched. Finally, the PDMS could be bonded to a glass slide with size of 75 mm × 25 mm × 2 mm after a surface oxygen plasma (PCE-6, Shenzhen, China) treatment at 29.6 W for 30 s. The height of the microfluidic channel was 120 µm. The width of the continuous flow was 160 µm, the same as the dispersed flow. The width of narrow area was 60 µm.

Formation of Fe^{3+}-TFP microcapsules

For the preparation of Fe^{3+}-TFP microcapsules, the Fe^{3+}-containing aqueous solution was firstly prepared by dissolving $FeCl_3 \cdot 6H_2O$ in a 2 wt% poly(vinyl alcohol) aqueous solution to the final concentration of 5 mM, and used as a continuous phase in the microfluidic chips, while the discontinuous oil phase was composed of 5 mM 1,3,5-triformylphloroglucinol (TFP) of o-xylene. The microcapsules were generated by first pumping the continuous water phase into the microfluidic chip at the speed of 3 µl min^{-1} to fill the appropriate channels, followed by the pump of discontinuous o-xylene phase into the middle channel of the microfluidic chip at the speed of 1.5 µL min^{-1}. To obtain smaller microcapsules, the water/oil flow rates were tuned, with the speed of 20 µl min^{-1} for water phase and 10 µl min^{-1} for oil phase. After the microcapsules were collected to a vial containing DI water, they were washed with DI water five times to remove any residual $FeCl_3$ and PVA surfactants. Finally, the formed microcapsules were stored in DI water for further study.

Fabrication of metal-TFP/PAN composite membranes

Metal-TFP composite membranes were prepared by in-situ interfacial self-assembly on PAN ultrafiltration membrane. Typically, the aqueous solution contain metal ions (M^{n+}) was poured onto the surface of PAN substrate clamped with a stainless steel ring. After being kept for 5 min, excess aqueous solution was decanted, and the residual liquid droplet on PAN substrate surface was further removed using a gas gun of N_2. Subsequently, TFP-containing o-xylene solution was poured onto the metal ions-absorbed membrane surface for a certain time to form metal-TFP active layer. Then, the organic solution was drained, and the membrane surface was rinsed with fresh n-hexane to remove any unreacted molecules and underwent a thermal treatment (50 °C, 5 min) in an oven to stabilize the generated metal-TFP structure. Finally, the obtained metal-TFP/PAN composite membranes were stored in DI water prior to permeation test.

Characterization methods

Transmission electron microscopy (TEM)

Transmission electron microscopy (TEM) images and energy dispersive X-ray spectroscopy (EDX) elemental maps were acquired using transmission electron microscope (Talos F200X G2, Thermo Fisher) with an operation voltage of 200 kV. For the freestanding film formed at free water-organic interface, the TEM sample was simply prepared by transferring nanofilms onto a 400 mesh copper grid. For the in-situ formed composite membrane, TEM samples were prepared as follows: the composite membrane was put into propylene carbonate (PC) and kept for at least 60 min to ensure that the PAN support was completely removed, and then the isolated metal-TFP nanofilm was transferred onto 400 mesh copper grid, followed by washing with DI water (immersion for 15 min) and dried at room temperature.

Atomic force microscopy (AFM)

Cypher ES (Asylum Research) and Dimension ICON (Bruker) atomic force microscopes were used to characterize metal-TFP based flat films and microcapsules, respectively. The morphology and thickness of samples was conducted in tapping mode with resolution of 512 points per line and a speed of 0.5-1 Hz. The Young's modulus of metal-TFP nanofilms was acquired with Cypher ES by using AM-FM model, which benefited of normal AC model (so called AM) with the high sensitivity of Frequency Modulation (FM) mode. The probe (AC 160) was used for the measurements. According to the Young's mapping and the corresponding statistical histogram, the Young's modulus value was derived using a Hertz Punch Model and fitted with Gauss model. Herein, poly(vinylidene fluoride) with a Young's modulus of 2.45 GPa was used as a standard sample to calibrate the radius of Hertz Punch Model. 'Gwyddion 2.58' software was used to process all the AFM images.

For freestanding films formed at free water-organic interface, AFM samples were simply prepared by transferring films onto silicon wafers and then washed with dichloromethane and water in sequence, and finally dried at room temperature. For microcapsules, AFM samples were prepared as follows: the microcapsule suspensions (5 μL) was dropped onto silicon wafers and allowed to air dry, and then carefully washed with dichloromethane (DCM) to remove remaining TFP released from the microcapsules and air-dried again. For the in-situ formed composite membrane, AFM samples were prepared as follows: the composite membrane was put into PC and kept for at least 60 min to ensure that the PAN support was completely removed, and then the isolated metal-TFP nanofilm was transferred on silicon wafer and dried at room temperature.

Scanning electron microscopy (SEM)

The surface and cross-section morphologies of metal-TFP nanofilms in-situ formed on PAN support membrane were analyzed by field emission scanning electron microscope (Regulus 8230, Hitachi) at an accelerating voltage of 5 kV. For cross-sectional study, composite membranes were prepared by soaking in ethanol and then freeze-fractured in liquid nitrogen and dried in dry air. All the samples were previously sputter coated with an ultrathin platinum layer (3 nm) by high vacuum coating instrument (Leica, EM ACE600) to avoid charging effects.

Ultraviolet-visible (UV-Vis) spectra

Ultraviolet-visible spectroscopy (UV-2700, Shimadzu) was applied to analyze the coordination between metal ion (Fe^{3+}, Sc^{3+}, and Cu^{2+}) and TFP. All the UV-Vis spectra were measured in ethanol or N,N-dimethylformamide (DMF) at the concentration of 0.1 mM.

Attenuated total reflectance infrared spectroscopy (ATR-IR)

The attenuated total reflectance infrared spectroscopy (ATR-IR) spectra of metal-TFP film were obtained on a Nicolet iS50 spectrometer in the range of 4000-400 cm^{-1} with 64 scans and a resolution of 4 cm^{-1}. To prepare ATR-IR sample, the metal-TFP film was isolated from the free water/oil interface by using an aluminum

foil as a collector. Then the collected film was washed with DCM and water respectively, and dried at 40 °C under vacuum. A piece of the same clean aluminum foil was used as the background.

X-ray photoelectron spectroscopy (XPS)

Surface elemental composition of membranes was examined by XPS (ESCALAB Xi+, Thermo Fisher). XPS peak positions were calibrated with the help of the C 1s peak at 284.8 eV. The XPS sample preparation procedure was the same as the AFM as mentioned above.

Raman spectra

The Raman spectrum of the metal-TFP naofilm was obtained by Raman spectrometer (Alpha300R, WITec) with a laser wavelength of 532 nm (semiconductor laser) and a spectral acquisition range of 50-3900 cm^{-1}. The Raman sample was prepared by transferring the freestanding metal-TFP nanofilm on to a clean glass slide.

Zeta potential

Zeta potential of Metal-TFP composite membrane surface was determined through measuring surface streaming potential with an electrokinetic analyzer (SurPass 3, Anton Paar GmbH) using 1 mmol L^{-1} KCl aqueous solution at 25.0 °C.

X-ray diffraction (XRD)

XRD experiments were performed on Bruker powder X-ray diffractometer (D8 Advance, Bruker) using Cu Kα radiation (40 kV, 40 mA) at room temperature. For XRD sample preparation, metal-TFP nanofilms were obtained by the reaction of 150 mL 5 mM metal salts ($FeCl_3$, $ScCl_3$, $CuCl_2$) in water with 150 mL 6 mM TFP in o-xylene with strong shaking for 30 min and filtrated with nonwoven fabrics. Then, the collected nanofilms were washed with water and DCM in sequence before dried in a vacuum oven at 60 °C overnight.

Density

The density of metal-TFP nanofilm was measured using helium pycnometry (AccuPyc II 1340, micromeritics). The density sample preparation procedure was similar to that of XRD as described above.

Evaluation of membrane separation performance

The membrane separation performance (including water permeance and salt rejection) of different metal-TFP composite membranes was evaluated in a cross-flow filtration system with an effective filtration area of 22.1 cm^2 at a cross-flow flux of approximately 150 L h^{-1}. All filtration tests were conducted at 5.0 bar using a 1000 ppm salt (NaCl and Na_2SO_4) aqueous solution at 25 ± 2 °C and pH of 7.5 ± 0.2. Membranes were stabilized for at least 4 h with feed solution before testing. The water permeance (P) and salt rejection (R) were calculated according to Eqs. (1) and (2), respectively:

$$P = \frac{V}{S \times \Delta P \times \Delta t} \qquad (1)$$

$$R\ (\%) = \left(1 - \frac{C_P}{C_F}\right) \times 100\% \qquad (2)$$

where V (L) was permeate volume, S (m^2) was the effective filtration area of the membrane (22.1 cm^2), ΔP (bar) was the operation pressure (bar), Δπ (bar) was the osmotic pressure

differential across the membrane (bar), Δt (h) was the time to collect V, C_P and C_F were the salt concentration of the permeate and feed solutions, respectively. In this work, the salt concentration was estimated using a conductivity meter (FE38, Mettler-Toledo)

Theoretical Calculations

Binding energy caculation

The binding energy between transition metal ions (Fe^{3+}, Sc^{3+}, and Cu^{2+}) and TFP was carried out with Gaussian 16 software[1] through the first-principles density functional theory (DFT) approach. All molecules involved in this study were firstly searched via Molclus software[2] and 10 geometries were produced. And then, xtb program[3] was employed to perform the pre-optimization and the six geometries were obtained in this step. These geometries were further optimized via Gaussain 16 software at the level of B3LYP/def2svp em=gd3bj and the frequency were also calculated at the same level to prevent the imaginary frequency. The single point

energy was calculated at the level of B3LYP/def2tzvp em=gd3bj and the solvent effect was adopted SMD implicit solvent[4]. The binding energy was calculated via the following formula:

$\Delta E_{binding}=E_{(metal\text{-}TFP)}-E_{(metal\ ion)}-E_{(TFP)}$

Calculation of monomer partition coefficients in different solvent systems

The partition ratio K of a solute in two solvents could be caculated from the difference in free energy of the solute in the two solvents (Equation S1):

$\Delta G = -RT \ln K$ (S1)

K could ve expressed in the form of the distribution coefficient logP (Equation S2):

$$\log P_{A/B} = -\frac{\Delta G_{solv}(A) - \Delta G_{solv}(B)}{2.303RT} = -\frac{E(A) - E(B)}{2.303RT}$$

where, $\log P_{A/B}$ was the partition coefficient of the solute between solvent A and solvent B, and ΔGsolv and E were the free energy and single point energy of the solute in the corresponding solvent, respectively. The geometries were optimized via gaussian 16 software at the theory of B3LYP combined with basis of def2svp, and the frequency were also calculated at the same level to prevent the imaginary frequency. The single point energies were calculated at the theory of B3LYP combined with basis of def2tzvp. Moreover, the self-consistent reaction field (SCRF) with using the SMD model and the dispersion correction method (DFT-D3) were also used during all the calculations.

Model for polymer framework

The amorphous metal-TFP supramolecular polymer models were constructed by using molecular dynamic (MD) simulation based on Materials Studio 2019 software with the Compass II force field. The amorphous cell module was used for the construction of the amorphous box with the cell dimensions of 29 Å. 100 TFP and 100 metal ions were placed for each system, and the initial density was set in the range of 2.0-3.0 g cm^{-3}. The initial structure was optimized via the Forcite module. MD simulations were then performed in NPT and NVT ensembles for 50ps, respectively. After the pre-balance, finally MD simulation was performed in NPT ensembles for 1000 ps The temperature (298.15 K) and pressure (1 bar) were controlled via a Nose-Hoover thermal bath

and Berendsen pressure bath, respectively. The integration step length was set to 1 fs and the long-range interaction cutoff radius was set to 1.2 nm. Structural information such as the pore size distribution was calculated by the ZEO++ software package[5] based on the Voronoi decomposition.

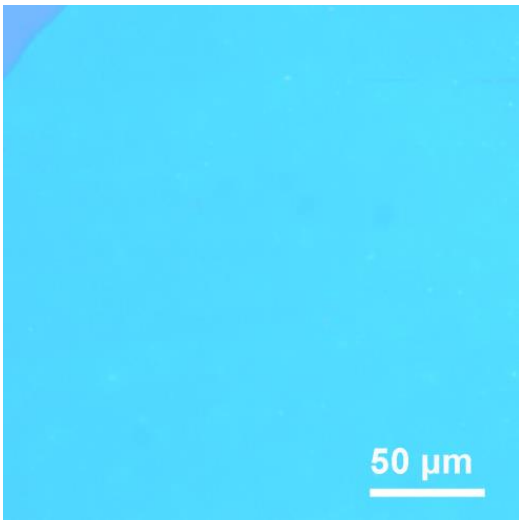

Figure S1. Optical microscopy images of freestanding Fe^{3+}-TFP nanofilm transferred onto SiO_2/Si substrate.

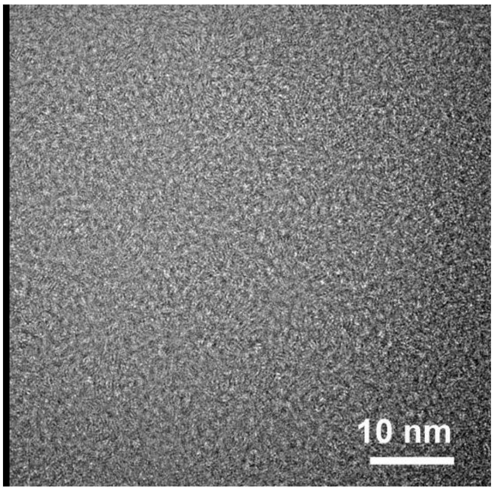

Figure S2. High-resolution TEM image of freestanding Fe^{3+}-TFP nanofilm formed at the free water/o-xylene interface.

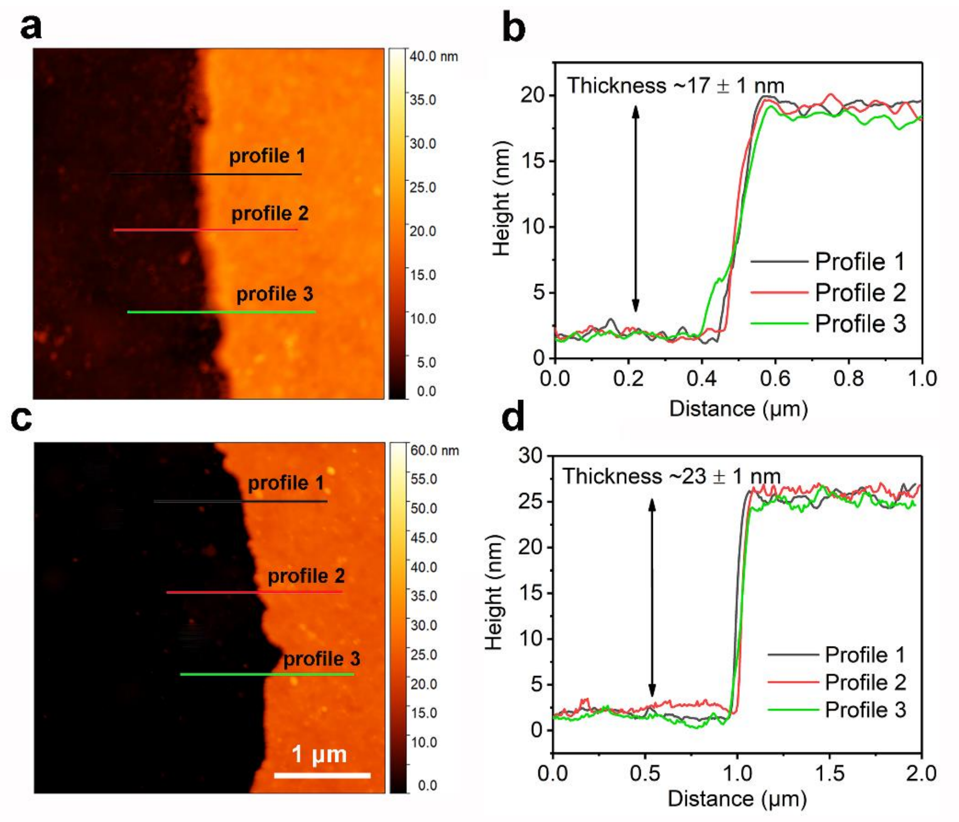

Figure S3. AFM height image and corresponding height profile of freestanding Fe^{3+}-TFP nanofilms formed at the free water/o-xylene interface with different assembly time. (a and b) 5 min; (c and d) 10 min.

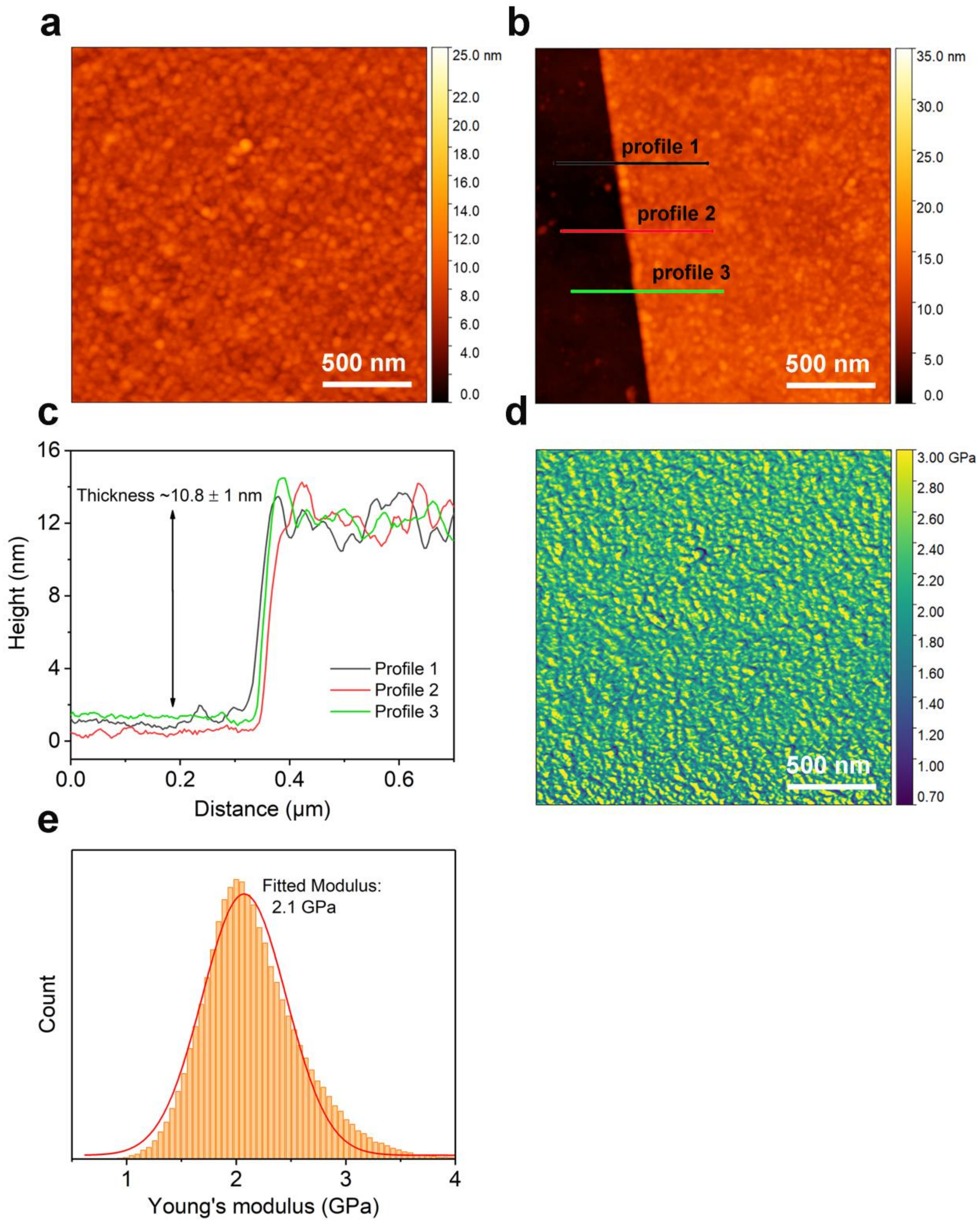

Figure S4. AFM characterization of freestanding Sc^{3+}-TFP nanofilms formed at the free water/o-xylene interface. (a-c) AFM topography image (a), AFM height image (b) and corresponding height profile (c) of Sc^{3+}-TFP nanofilms transferred onto silicon wafers. (d and e) Young's modulus mapping (d) and the corresponding statistical histogram (e) of the Sc^{3+}-TFP nanofilm transferred onto the silicon wafer.

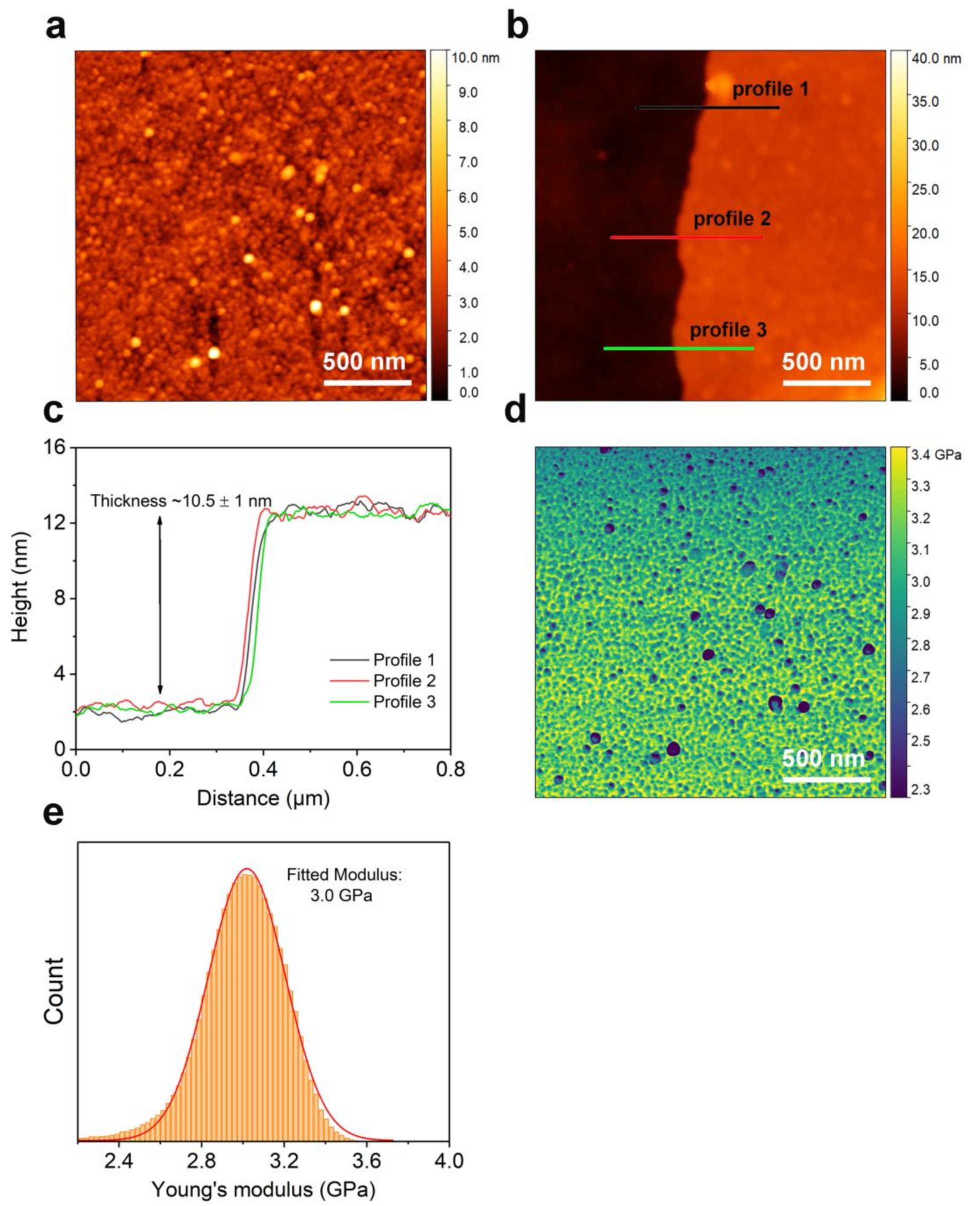

Figure S5. AFM characterization of freestanding Cu^{2+}-TFP nanofilms formed at the free water/o-xylene interface. (a-c) AFM topography image (a), AFM height image (b) and corresponding height profile (c) of Cu^{2+}-TFP film transferred onto silicon wafers. (d and e) Young's modulus mapping (d) and the corresponding statistical histogram (e) of the Cu^{2+}-TFP film transferred onto the silicon wafer.

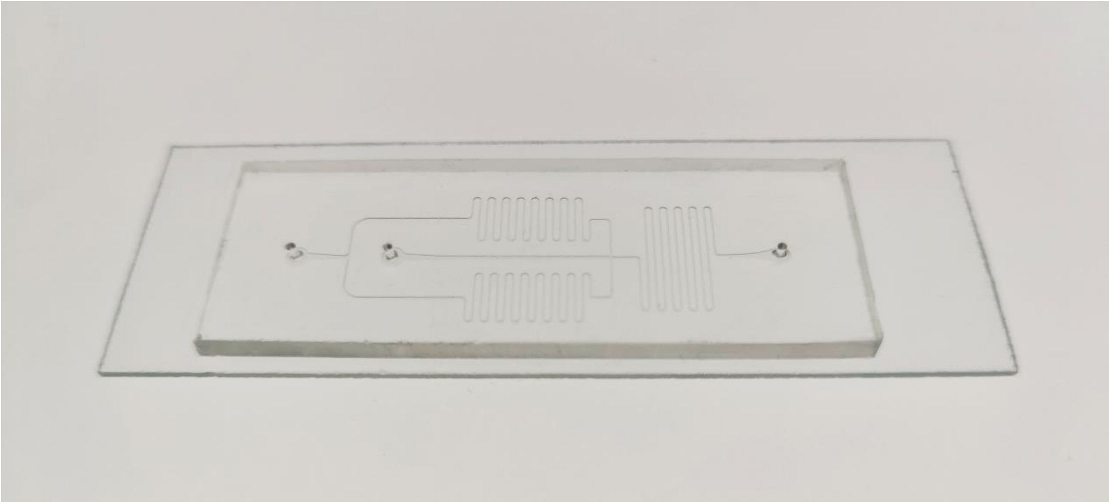

Figure S6. Photoshop of the Microfluidic chip.

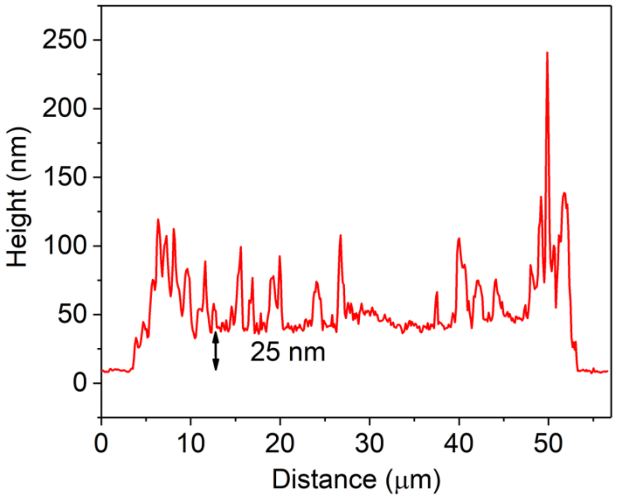

Figure S7. AFM height profile of the dried Fe^{3+}-TFP microcapsule transferred onto silicon wafers.

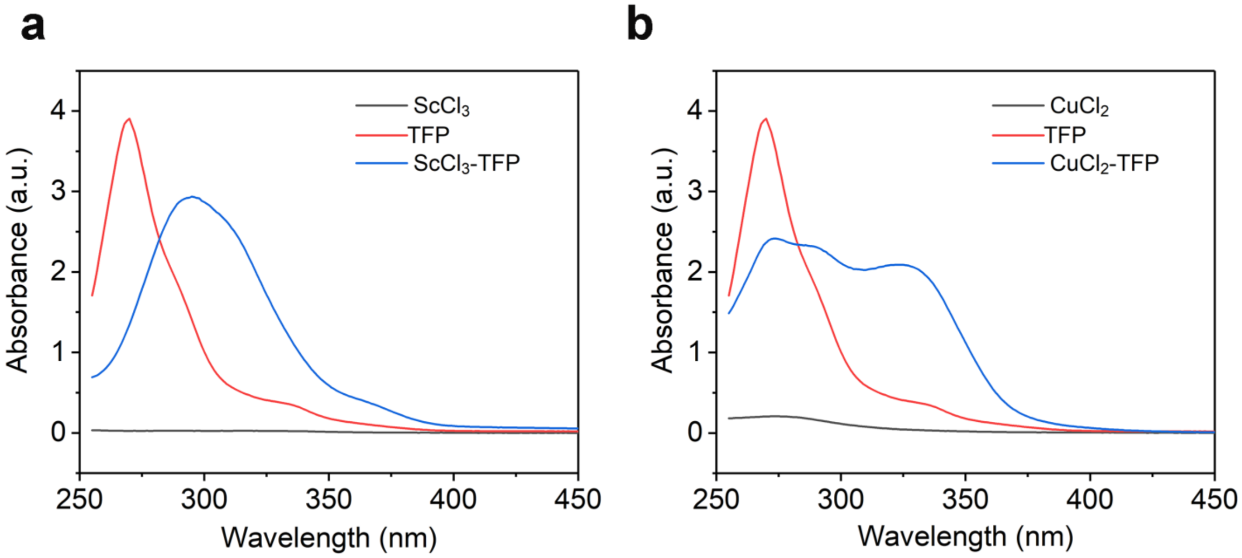

Figure S8. UV-Vis spectra of TFP ligand with different metal ions. (a) TFP, $ScCl_3$ and their complex. (b) TFP, $CuCl_2$ and their complex. All the UV-Vis spectra were measured in ethanol at the concentration of 0.1 mM.

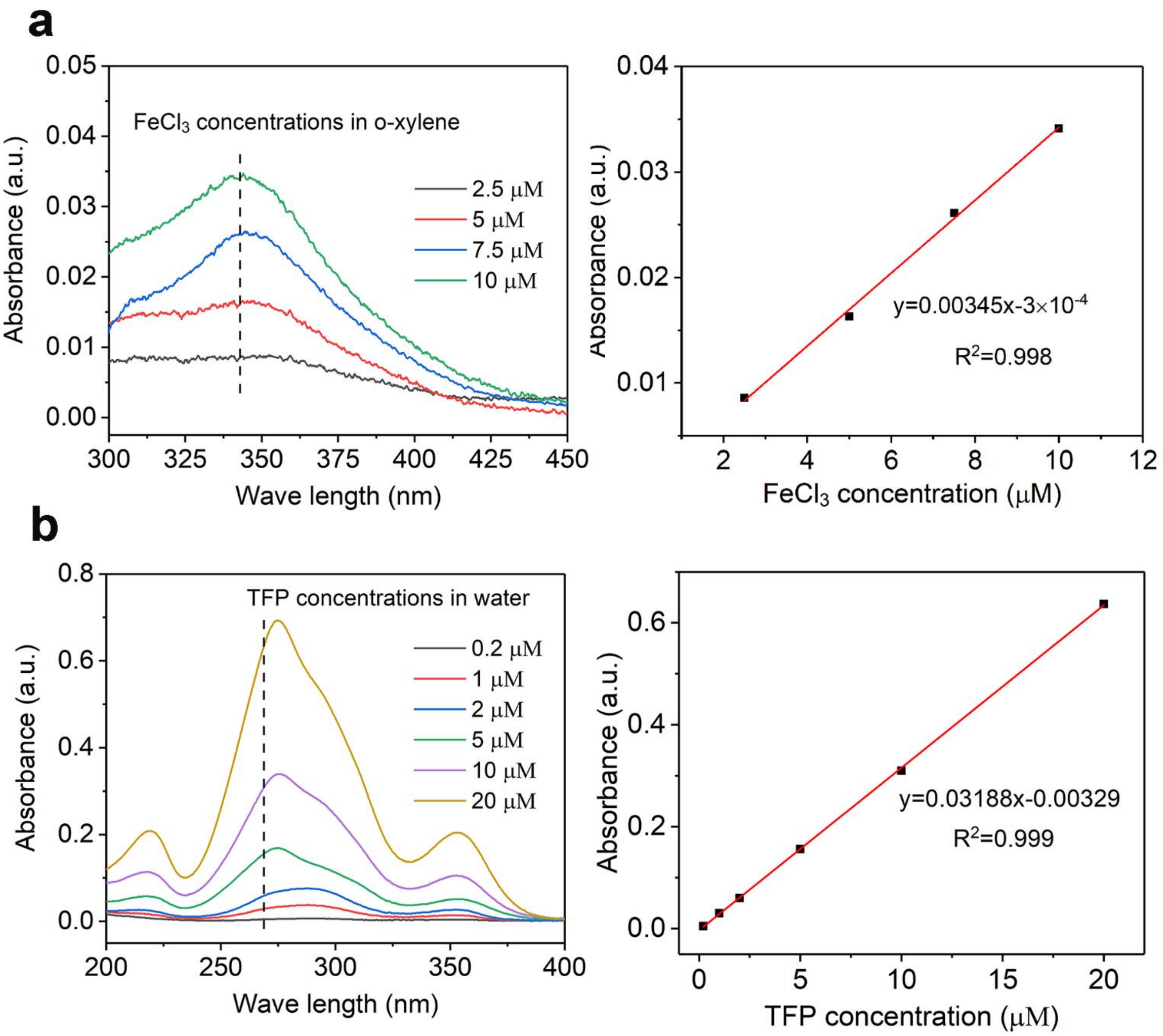

Figure S9. (a and b) UV absorption peak and absorbance versus concentration standard curve of $FeCl_3$ in o-xylene, TFP in water, respectively.

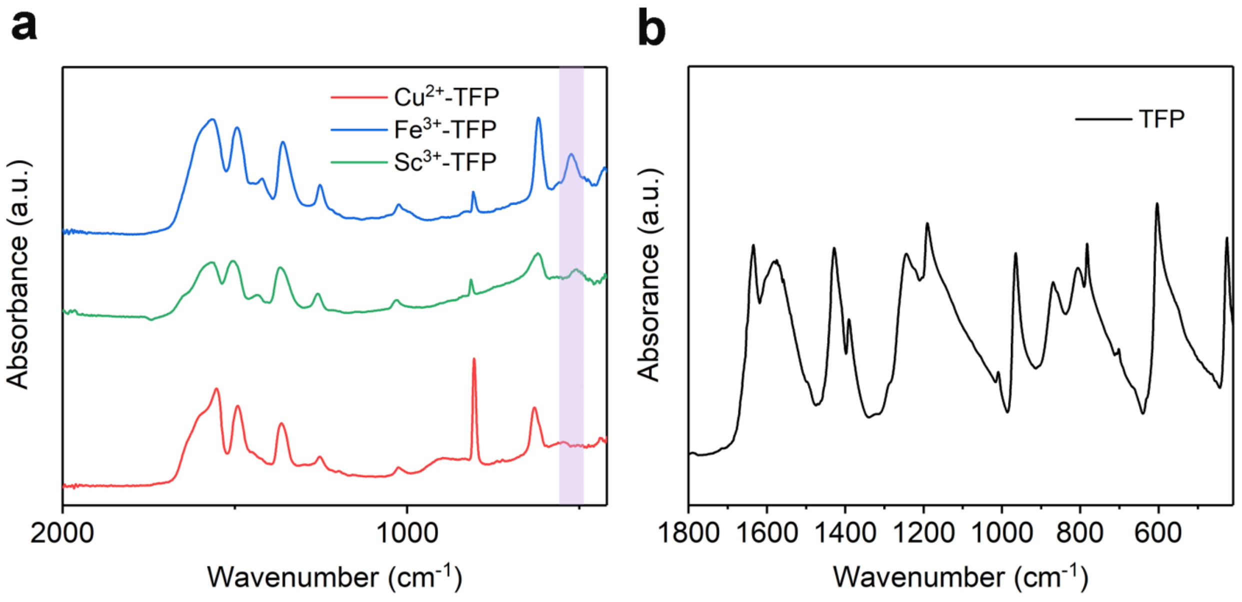

Figure S10. ATR-IR spectra. (a and b) ATR-IR spectra of freestanding metal-TFP nanofilms formed at free water/o-xylene interface (a) and TFP (b).

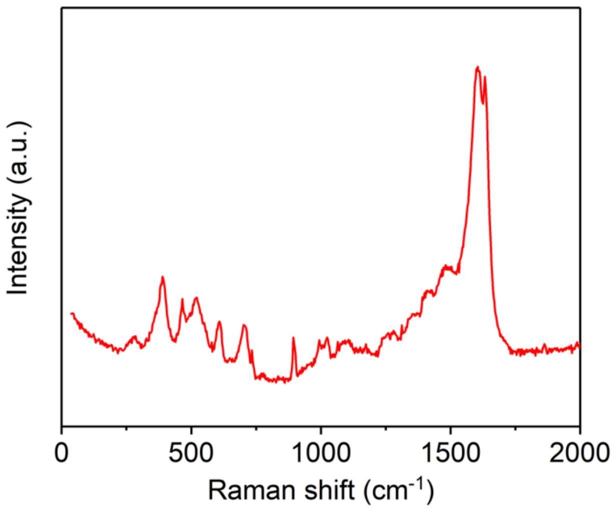

Figure S11. Raman spectrum of freestanding Fe^{3+}-TFP nanofilm formed at free water/o-xylene interface.

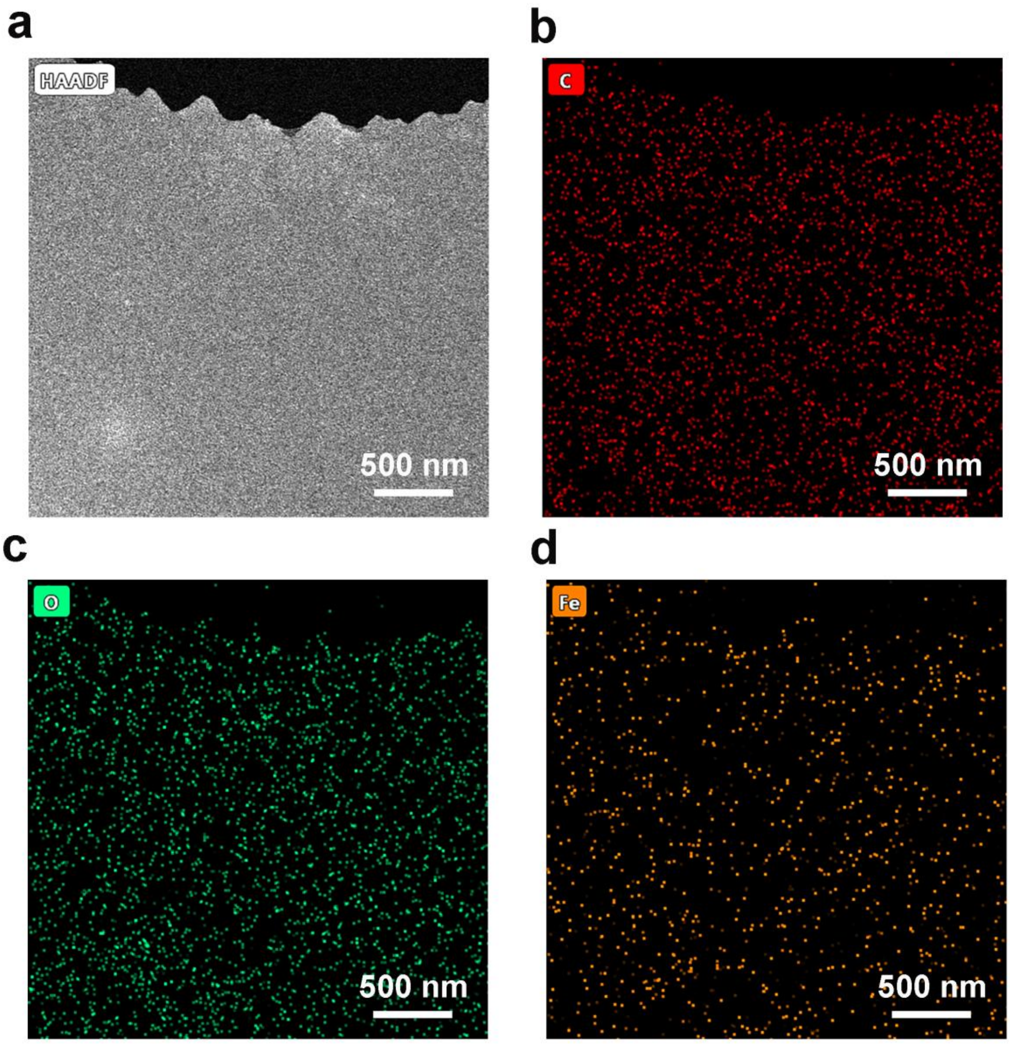

e

Z	Element	Family	Atomic Fraction (%)	Atomic Error (%)
6	C	K	67.30	7.99
8	O	K	30.17	7.00
26	Fe	K	2.53	0.46

Figure S12. STEM characterization of freestanding Fe^{3+}-TFP nanofilm formed at free water/o-xylene interface**.** HAADF-STEM image (a) and elemental maps of C (b), O (c), Fe (d), and the atomic composition (e) of Fe^{3+}-TFP nanofilm formed at free water/o-xylene interface.

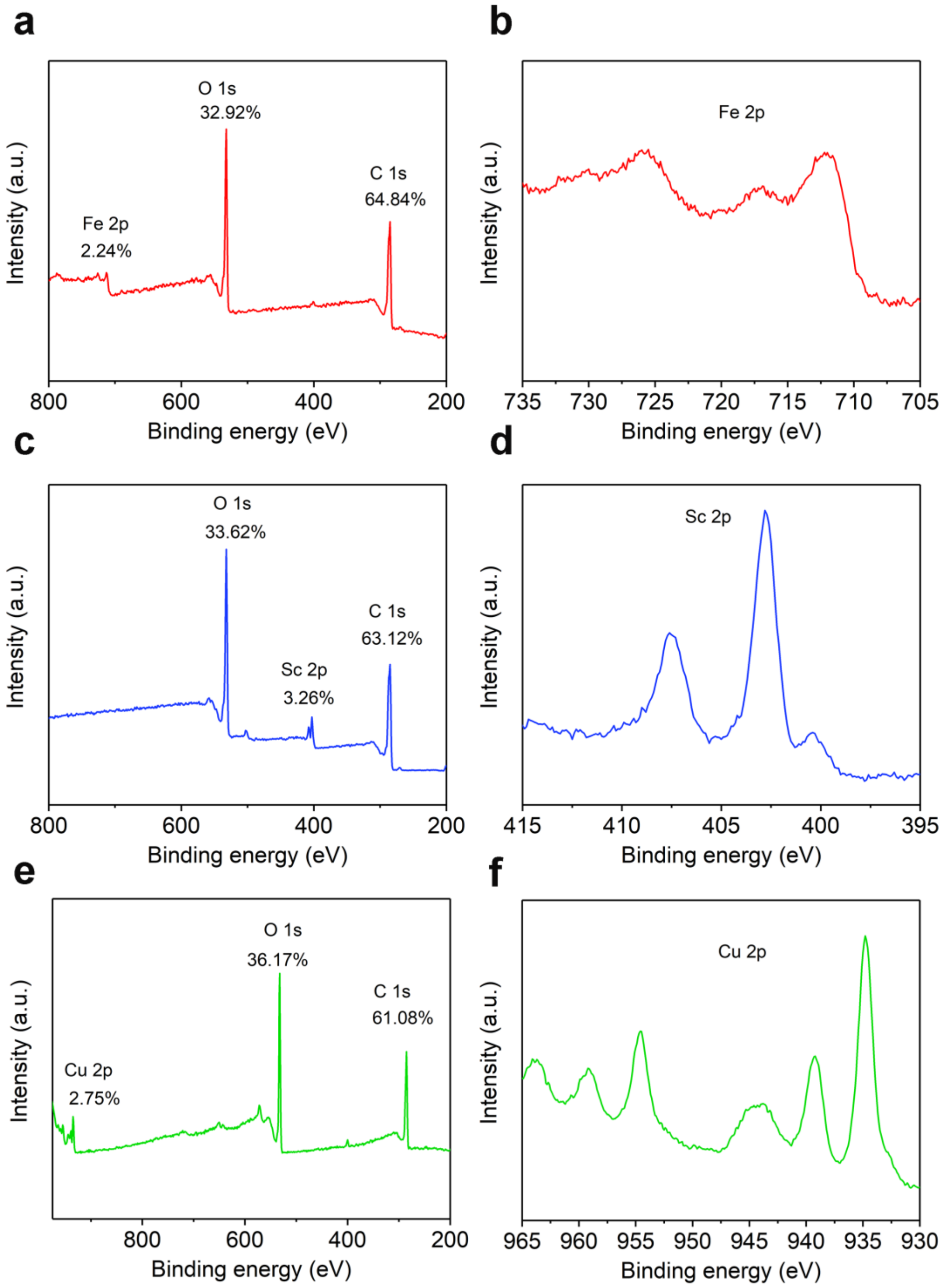

Figure S13. XPS spectra of freestanding metal-TFP nanofilms formed at free water/o-xylene interface. (a and b) Survey spectrum (a) and Fe 2p core level spectrum (b) of Fe-TFP nanofilms. (c and d) Survey spectrum (c) and Sc 2p core level spectrum (d) of Sc-TFP nanofilms. (e and f) Survey spectrum (e) and Cu 2p core level spectrum (f) of Cu-TFP nanofilms.

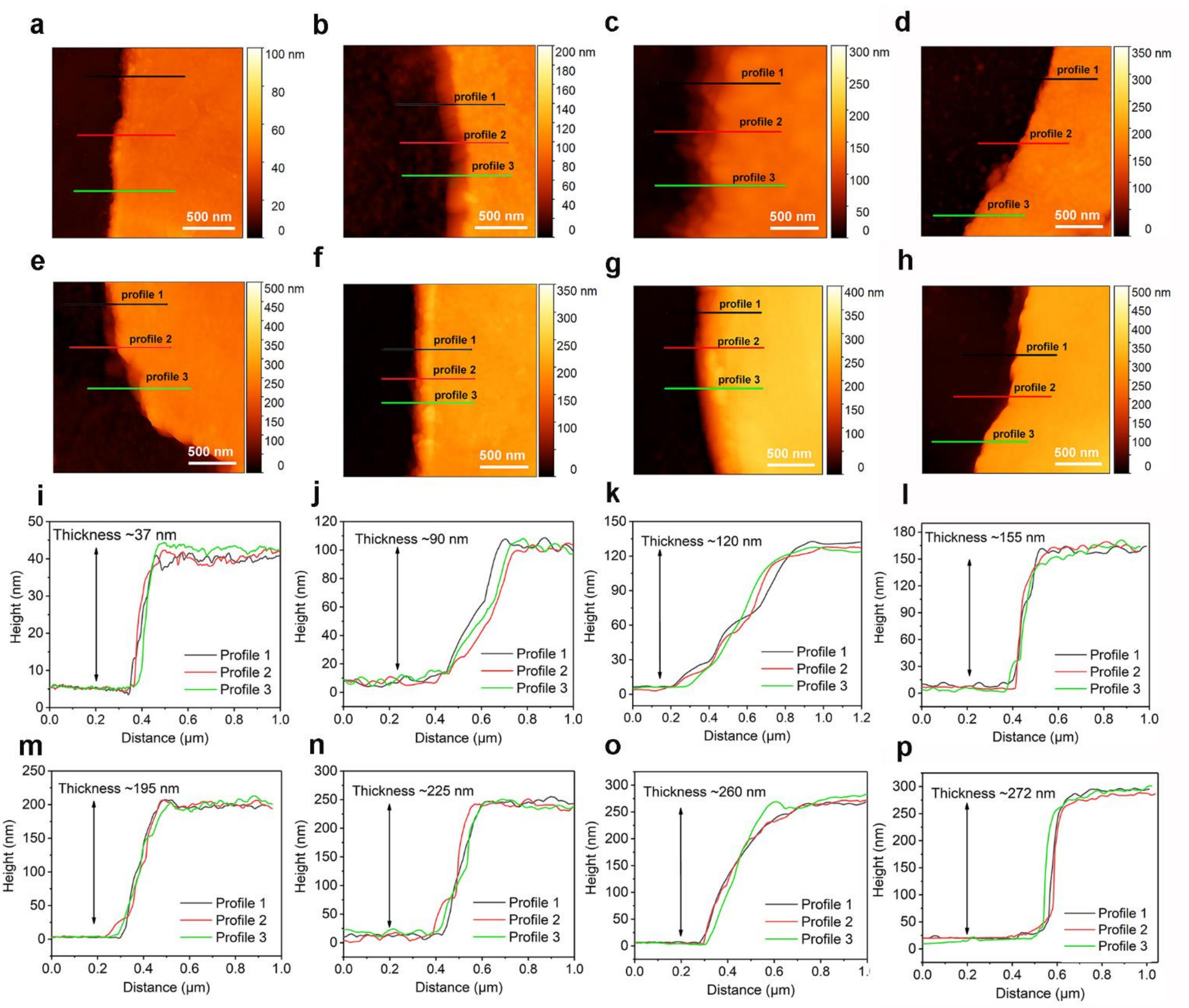

Figure S14. AFM height images and the corresponding height profiles of isolated Fe^{3+}-TFP active layers. The active layers were prepared via the in-situ self-assembly method with different assembly time on the PAN substrate and then transferred the isolated active layers onto silicon wafers. (a and i) 0.5 min. (b and j) 2 min. (c and k) 3.5 min. (d and l) 5 min. (e and m) 8 min. (f and n) 10 min. (g and o) 20 min. (h and p) 30 min.

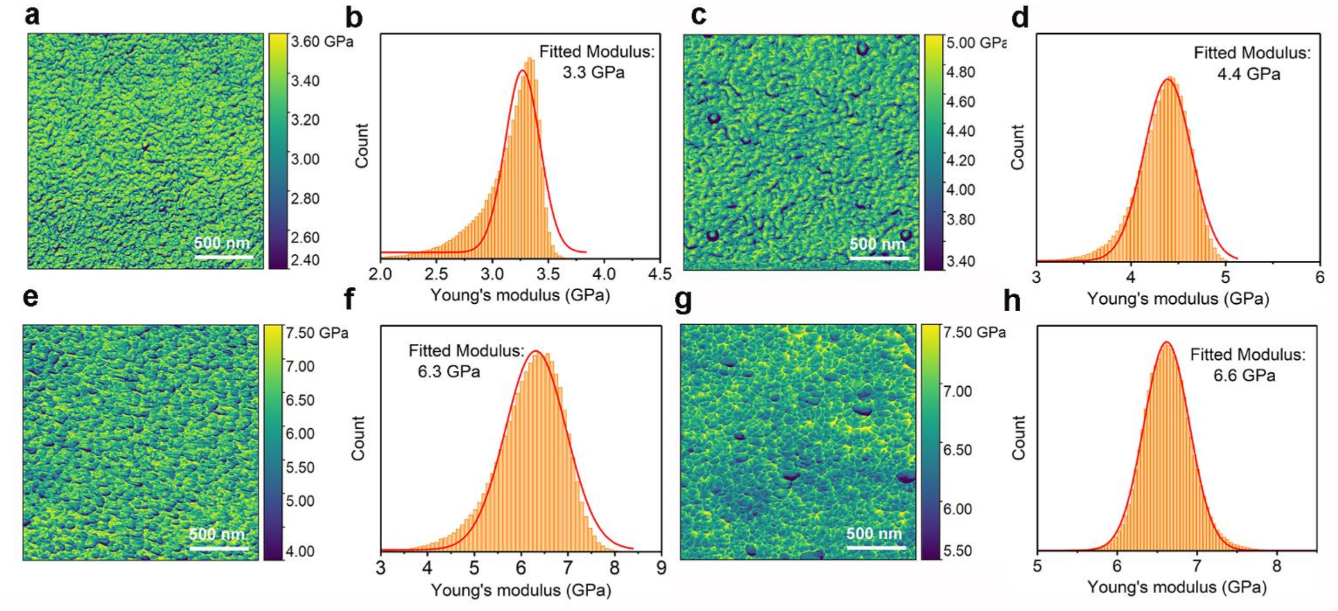

Figure S15. Young's modulus mappings and the corresponding statistical histograms of Fe^{3+}-TFP active layers. The active layers were prepared via the in-situ self-assembly method with different assembly time on the PAN substrate and then transferred the isolated active layers onto silicon wafers. (a and b) 0.5 min. (c and d) 3.5 min. (e and f) 5 min. (g and h) 10 min.

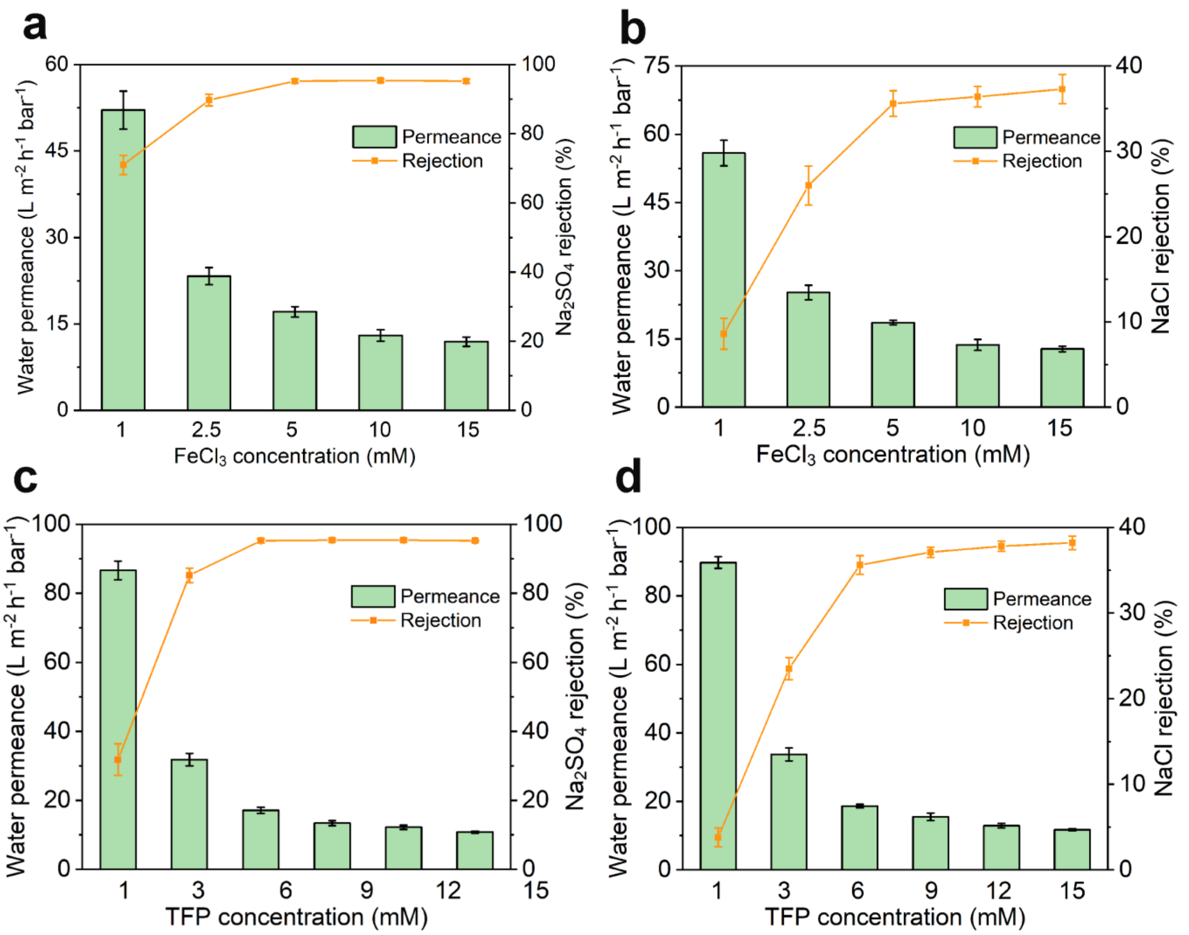

Figure S16. Separation performance of Fe^{3+}-TFP/PAN composite membranes. (a and b) Water permeance and salt rejection of Fe^{3+}-TFP/PAN composite membranes with different $FeCl_3$ concentration for separating Na_2SO_4 (a) and NaCl (b) aqueous solutions, TFP concentration and assembly time were fixed at 6 mM and 5 min, respectively. (c and d) Water permeance and salt rejection of Fe^{3+}-TFP/PAN composite membranes with different TFP concentration for separating Na_2SO_4 (c) and NaCl (d) aqueous solutions, $FeCl_3$ concentration and assembly time were fixed at 5 mM and 5 min, respectively. All membranes were tested with 1000 ppm salt aqueous solution under 5.0 bar, 25 ± 2 °C and pH 7.5 ± 0.2.

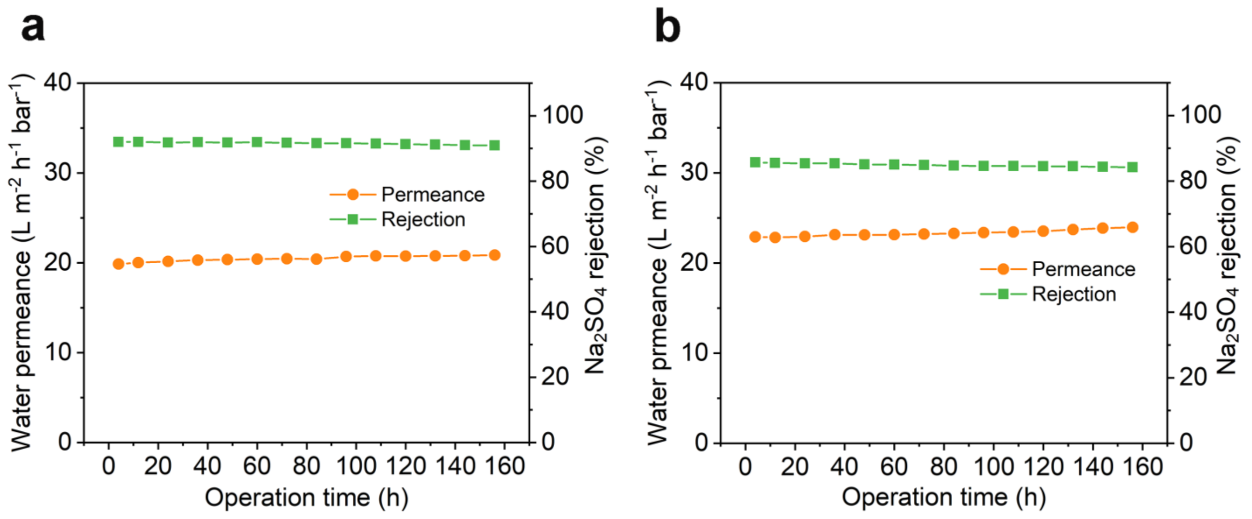

Figure S17. Stability of metal-TFP/PAN composite membranes. (a and b) Long-term operation stability of Sc^{3+}-TFP/PAN composite membrane (a) and Cu^{2+}-TFP/PAN composite membrane (b). All membranes were tested with 1000 ppm Na_2SO_4 aqueous solution under 5.0 bar, 25 ± 2 °C and pH 7.5 ± 0.2.

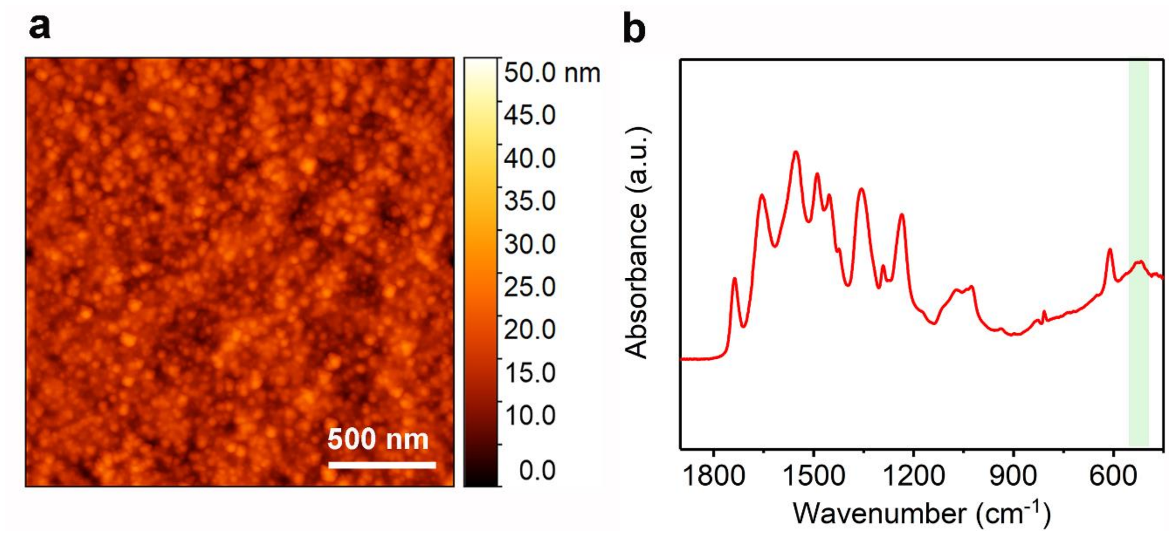

Figure S18. (a) AFM topology of isolated Fe^{3+}-TFP active layer after nanofiltration test. (b) ATR-IR spectra of Fe^{3+}-TFP/PAN composite membrane after nanofiltration test.

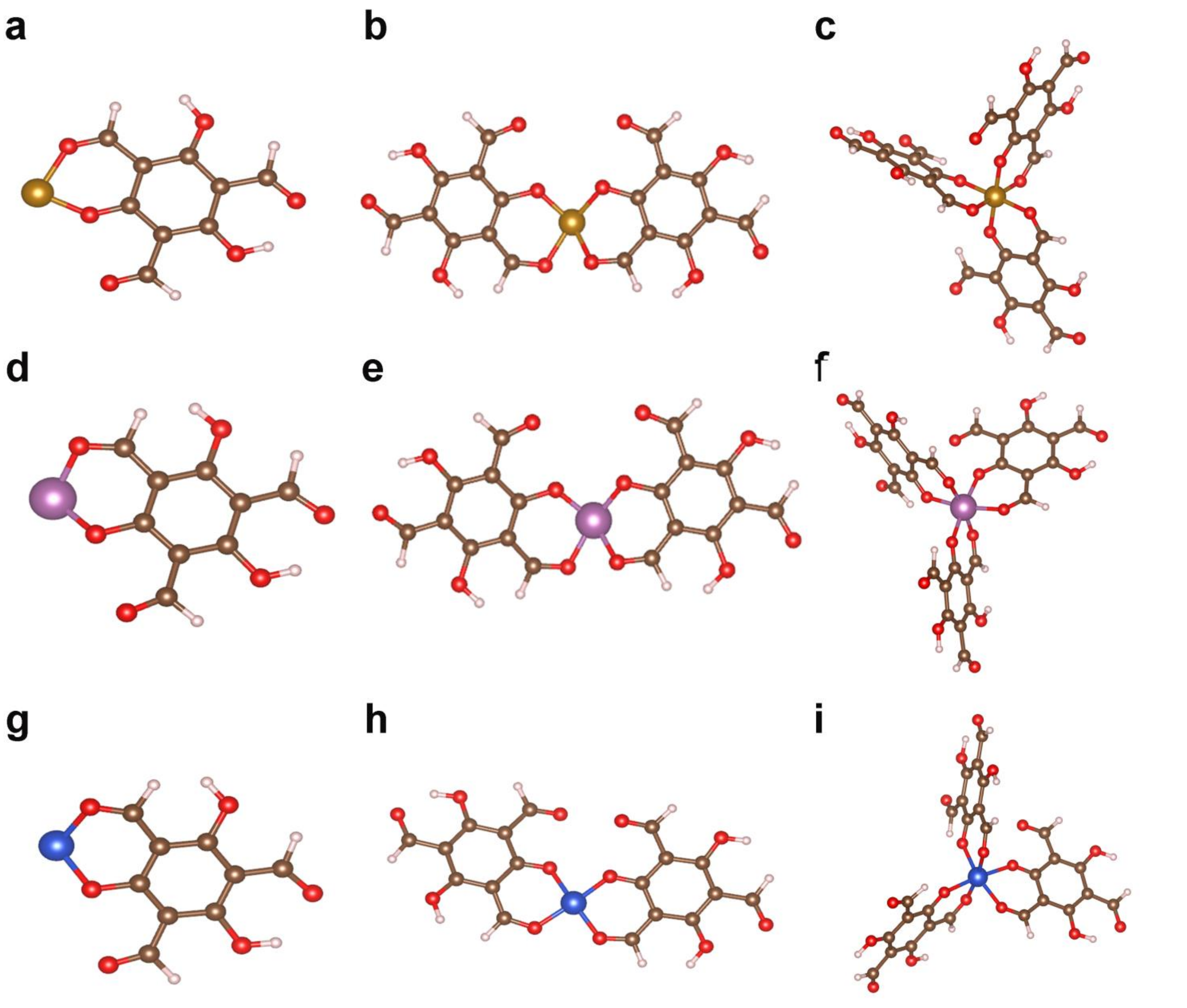

Figure S19. The stimulated complexes of TFP with different metal ions through DFT calculation. (a-c) Mono- (a), bis- (b), tris- (c) complex of Fe^{3+} with TFP. (d-f) Mono- (d), bis- (e), tris (f) -complex of Sc^{3+} with TFP. (g-i) Mono- (g), bis- (h), tris (i) -complex of Cu^{2+} with TFP.

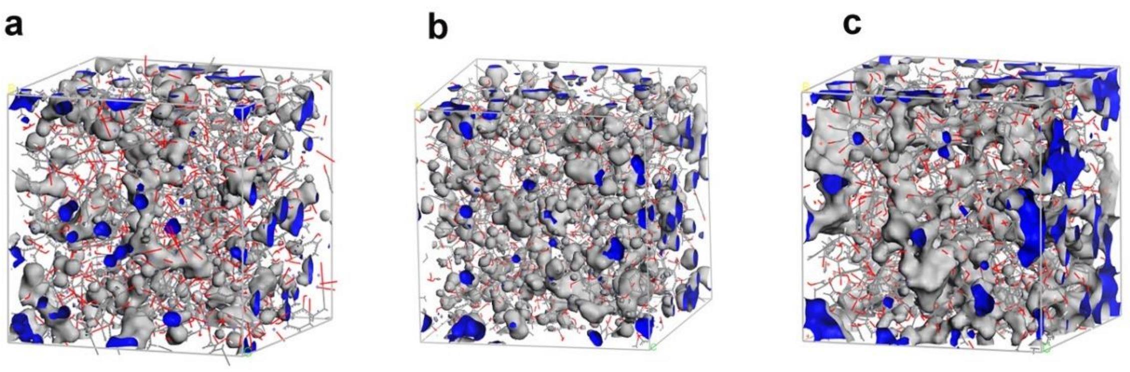

Figure S20. Three-dimensional view of an amorphous cell containing cross-linked metal-TFP network with a size of 29 Å × 29 Å × 29 Å. (a) Fe^{3+}-TFP. (b) Sc^{3+}-TFP. (c) Cu^{2}+-TFP.

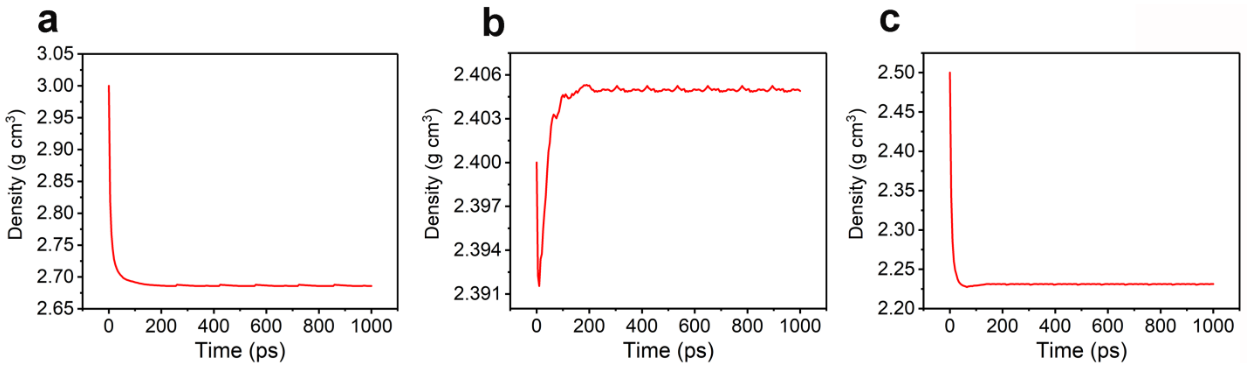

Figure S21. Theoretic density variation of each simulated framework with respect to time. (a) Fe^{3+}-TFP. (b) Sc^{3+}-TFP. (c) Cu^{2}+-TFP.

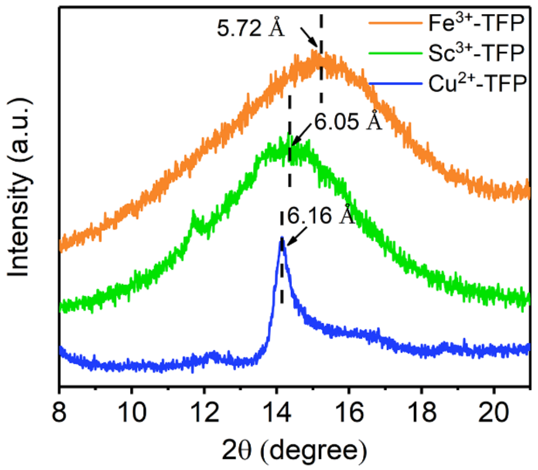

Figure S22. XRD characterization of isolated metal-TFP active layers.

Table S1. The partition coefficient of monomers between different solvents

Monomer	Solvent	E (Hartree)	$logP_{o\text{-}xylene/water}$
$FeCl_3$	O-xylene	-2644.59746	-8.15
	Water	-2644.6149	
TFP	O-xylene	-762.575262	3.43
	Water	-762.56793	

Table S2. Water permeance and corresponding membrane thickness with the different assembly time.

Assembly time (min)	Water permeance (A, Lm^{-2} h^{-1} bar^{-1})	Mn^{+}-TFP active layer thickness (δ, nm)	Constant permeability=A*δ ($\times 10^{-8}$ L m^{-1} h^{-1} bar)
0.5	62.9±3.5	37	2.694×10^{-6}
2	31.7±1.4	90	
3.5	21.5±0.9	120	
5	17.1±0.9	155	
8	14.6±0.7	195	
10	12.3±0.5	225	

Table S3. Comparision of Fe^{3+}-TFP composite membranes with the state of the art nanofiltration membranes reported in the literature.

	Nanofiltration membranes	Permeance ($L\ m^{-2}\ h^{-1}\ bar^{-1}$)	Na_2SO_4 rejection (%)	Feed salt concentration	Operation pressure (bar)	Ref.
	Fe^{3+}-TFP	21.5	95.0	1000 ppm	5	**Current work**
GO	GO+9.1wt% TBO	0.41	90.0	0.01 mM	50	[6]
	r-hGO	7.3	98.5	2000 ppm	4	[7]
	GO@nylon6-16	3.7	57.0	20 mM	1	[8]
	GO/Cellulose	8	67.0	10 mM	-	[9]
	brGO	3.26	58.0	20 mM	5	[10]
	PVDF-PAA/GO	2.8	79.0	2000 ppm	4	[11]
	GO/PAN	5.2	68.0	950 ppm	20.7	[12]
	GO/PSF	16	45.0	20 mM	0.34	[13]
Metal-TA	Fe^{3+}-TA/PAN	40.9	15.0	1000 ppm	2	[14]
	Fe^{3+}-TA/PAN-COOH	13.6	90.2	1000 ppm	5	[15]
	TA-$Fe(acac)_3$/PES	8.6	97.3	0.01M	5	[16]
	Ti^{4+}-TA/PSF	8.5	70.3	5 mM	2	[17]
	Fe^{3+}-TA/PES	23	62.1	1000 ppm	2	[18]
COF	Polyimine COF	0.74	71	2000 ppm	15.5	[19]
	IISERP-COOH-COF1	0.5	96.3	2000 ppm	2	[20]
	Bi-layered COF	0.57	95.7	1000 ppm	3	[21]
	TpHz COF	4.05	58.3	1000 ppm	0.4	[22]
MOF	MOF UiO-66	0.14	98.0[a]	2000 ppm	10	[23]
	ZIF-8/TA/PES	3.6	92.2	1000 ppm	5	[24]
	ZIF-8/TA-Zn^{2+}/PES	5.1	93.6	2000 ppm	5	[25]
	MOF-303	0.74	96.0	0.1 wt%	5	[26]
PA-TC membranes	Commercial Dow NF 270	13.2	98	2000 ppm	4.8	[27]
	Commercial Dow NF 90	6.7	98.6	2000 ppm	4.8	[27]
	HZNc-PA	12.2	94.7	1000 ppm	6	[28]
	PSF-G4D-1-PA	26.4	99.1	2000 ppm	10	[29]
	PA with turing structure	24.8	99.6	2000 ppm	4.8	[30]
	BFS-based PA	26.5	96.6	1000 ppm	6	[31]
	NaCl@PIP PA	16.6	97.8	1000 ppm	5	[32]

References

[1] M. J. T. Frisch, G. W.; Schlegel, H. B.; Scuseria, G. E.; Robb, M. A.; Cheeseman, J. R.; Scalmani, G.; Barone, V.; Mennucci, B.; Petersson, G. A.; Nakatsuji, H.; Caricato, M.; Li, X.; Hratchian, H. P.; Izmaylov, A. F.; Bloino, J.; Zheng, G.; Sonnenberg, J. L.; Hada, M.; Ehara, M.; Toyota, K.; Fukuda, R.; Hasegawa, J.; Ishida, M.; Nakajima, T.; Honda, Y.; Kitao, O.; Nakai, H.; Vreven, T.; Montgomery, J. A., Jr.; Peralta, J. E.; Ogliaro, F.; Bearpark, M.; Heyd, J. J.; Brothers, E.; Kudin, K. N.; Staroverov, V. N.; Kobayashi, R.; Normand, J.; Raghavachari, K.; Rendell, A.; Burant, J. C.; Iyengar, S. S.; Tomasi, J.; Cossi, M.; Rega, N.; Millam, J. M.; Klene, M.; Knox, J. E.; Cross, J. B.; Bakken, V.; Adamo, C.; Jaramillo, J.; Gomperts, R.; Stratmann, R. E.; Yazyev, O.; Austin, A. J.; Cammi, R.; Pomelli, C.; Ochterski, J. W.; Martin, R. L.; Morokuma, K.; Zakrzewski, V. G.; Voth, G. A.; Salvador, P.; Dannenberg, J. J.; Dapprich, S.; Daniels, A. D.; Farkas, O.; Foresman, J. B.; Ortiz, J. V.; Cioslowski, J.; Fox, D. J. Gaussian 16, revision A.03; Gaussian Inc.: Wallingford, CT, **2017**.

[2] Tian Lu. Molclus program, 1.9.9.5. www.keinsci.com/research/molclus.html.

[3] S. Grimme, C. Bannwarth, P. Shushkov, *J. Chem. Theory Comput.* **2017**, 13, 1989.

[4] A. V. Marenich, C. J. Cramer, D. G. Truhlar, *J. Phys. Chem. B* **2009**, 113, 6378.

[5] M. Pinheiro, R. L. Martin, C. H. Rycroft, A. Jones, E. Iglesia, M. Haranczyk, *J. Mol. Graph. Model.* **2013**, 44, 208.

[6] Z. Wang, C. Ma, C. Xu, S. A. Sinquefield, M. L. Shofner, S. Nair, *Nat. Sustain.* **2021**, 402-408.

[7] X. Chen, Z. Feng, J. Gohil, C. M. Stafford, N. Dai, L. Huang, H. Lin, *ACS. Appl. Mater. Interfaces* **2020**, 12, 1387.

[8] L. Chen, Y. Li, L. Chen, N. Li, C. Dong, Q. Chen, B. Liu, Q. Ai, P. Si, J. Feng, L. Zhang, J. Suhr, J. Lou, L. Ci, *Chem. Eng. J.* **2018**, 345, 536.

[9] G. Liu, H. Ye, A. Li, C. Zhu, H. Jiang, Y. Liu, K. Han, Y. Zhou, *Carbon* **2016**, 110, 56.

[10] Y. Han, Z. Xu, C. Gao, *Adv. Funct. Mater.* **2013**, 23, 3693.

[11] F. Baskoro, C.-B. Wong, S. R. Kumar, C.-W. Chang, C.-H. Chen, D. W. Chen, S. J. Lue, *J. Membr. Sci.* **2018**, 554, 253.

[12] Y. Oh, D. L. Armstrong, C. Finnerty, S. Zheng, M. Hu, A. Torrents, B. Mi, *J. Membr. Sci.* **2017**, 541, 235.

[13] M. Hu, B. Mi, *Environ. Sci. Technol.* **2013**, 47, 3715.

[14] M. Jian, R. Qiu, Y. Xia, J. Lu, Y. Chen, Q. Gu, R. Liu, C. Hu, J. Qu, H. Wang, X. Zhang, *Sci. Adv.* **2020**, 6, eaay3998.

[15] D. Liu, Y. Chen, T. T. Tran, G. Zhang, *Sep. Purif. Technol.* **2021**, 2601, 118228.

[16] Y.-J. Shen, L.-F. Fang, Y. Yan, J.-J. Yuan, Z.-Q. Gan, X.-Z. Wei, B.-K. Zhu, *J. Membr. Sci.* **2019**, 587, 117146.

[17] H. Wu, J. Xie, L. Mao, *Sep. Purif. Technol.* **2020**, 233, 116051.

[18] L. Fan, Y. Ma, Y. Su, R. Zhang, Y. Liu, Q. Zhang, Z. Jiang, *RSC Advances* **2015**, 5, 107777.

[19] I. Gadwal, G. Sheng, R. L. Thankamony, Y. Liu, H. Li, Z. Lai, *ACS. Appl. Mater. Interfaces* **2018**, 10, 12295.

[20] C. Liu, Y. Jiang, A. Nalaparaju, J. Jiang, A. Huang, *J. Mater. Chem. A* **2019**, 7, 24205.

[21] A. Xiao, X. Shi, Z. Zhang, C. Yin, S. Xiong, Y. Wang, *J. Membr. Sci.* **2021**, 624, 119122.

[22] R. Wang, M. Wei, Y. Wang, *J. Membr. Sci.* **2020**, 604, 118090.

[23] X. Liu, N. K. Demir, Z. Wu, K. Li, *J. Am. Chem. Soc.* **2015**, 137, 6999.

[24] Y. Xu, Y. Xiao, W. Zhang, H. Lin, L. Shen, R. Li, Y. Jiao, B.-Q. Liao, *J. Membr. Sci.* **2021**, 618.

[25] Y. Xiao, W. Zhang, Y. Jiao, Y. Xu, H. Lin, *J. Membr. Sci.* **2021**, 624, 119101.

[26] S. Cong, Y. Yuan, J. Wang, Z. Wang, F. Kapteijn, X. Liu, *J. Am. Chem. Soc.* **2021**, 143, 20055.

[27] W. Fang, L. Shi, R. Wang, *J. Membr. Sci.* **2014**, 468, 52.

[28] Z. Sun, Q. Wu, C. Ye, W. Wang, L. Zheng, F. Dong, Z. Yi, L. Xue, C. Gao, *Nano Lett.* **2019**, 19, 2953.

[29] B. Yuan, S. Zhao, P. Hu, J. Cui, Q. J. Niu, *Nat. Commun.* **2020**, 11, 6102.

[30] Z. Tan, S. Chen, X. Peng, L. Zhang, C. Gao, *Science* **2018**, 360, 518.

[31] Y. Lu, R. Wang, Y. Zhu, Z. Wang, W. Fang, S. Lin, J. Jin, *Proc. Natl. Acad. Sci. USA* **2021**, 118, 37.

[32] L. Shen, R. Cheng, M. Yi, W. S. Hung, S. Japip, L. Tian, X. Zhang, S. Jiang, S. Li, Y. Wang, *Nat. Commun.* **2022**, 13, 500.
